# Measuring health equity in the ASEAN region: conceptual framework and assessment of data availability

**DOI:** 10.1186/s12939-023-02059-2

**Published:** 2023-12-05

**Authors:** Capucine Barcellona, Yzabel Bryanna Mariñas, Si Ying Tan, Gabriel Lee, Khin Chaw Ko, Savina Chham, Chhea Chhorvann, Borwornsom Leerapan, Nam Pham Tien, Jeremy Lim

**Affiliations:** 1 Saw Swee Hock School of Public Health, National University of Singapore, Singapore, Singapore; 2 Yale-NUS College, National University of Singapore, Singapore, Singapore; 3 Independent Consultant, Singapore, Singapore; 4 National Institute of Public Health Cambodia, Phnom Penh, Cambodia; 5 Faculty of Medicine Ramathibodi Hospital, Mahidol University, Bangkok, Thailand; 6 Hanoi University of Public Health, Hanoi, Vietnam

**Keywords:** Public Health, Health Equity, Data availability, ASEAN, Health systems, Global Health, Health disparities

## Abstract

**Background:**

Existing research on health equity falls short of identifying a comprehensive set of indicators for measurement across health systems. Health systems in the ASEAN region, in particular, lack a standardised framework to assess health equity. This paper proposes a comprehensive framework to measure health equity in the ASEAN region and highlights current gaps in data availability according to its indicator components.

**Methods:**

A comprehensive literature review was undertaken to map out a core set of indicators to evaluate health equity at the health system level. Secondary data collection was subsequently conducted to assess current data availability for ASEAN states in key global health databases, national health accounts, and policy documents.

**Results:**

A robust framework to measure health equity was developed comprising 195 indicators across Health System Inputs and Processes, Outputs, Outcomes, and Contextual Factors. Total indicator data availability equated to 72.9% (1423/1950). Across the ASEAN region, the Inputs and Processes sub-component of *Health Financing* had complete data availability for all indicators (160/160, 100%), while *Access to Essential Medicine* had the least data available (6/30, 20%). Under Outputs and Outcomes, *Coverage of Selected Interventions* (161/270, 59.63%) and *Population Health* (350/350, 100%) respectively had the most data available, while other indicator sub-components had little to none (≤ 38%). 72.145% (384/530) of data is available for all Contextual Factors. Out of the 10 ASEAN countries, the Philippines had the highest data availability overall at 77.44% (151/195), while Brunei Darussalam and Vietnam had the lowest data availability at 67.18% (131/195).

**Conclusions:**

The data availability gaps highlighted in this study underscore the need for a standardised framework to guide data collection and benchmarking of health equity in ASEAN. There is a need to prioritise regular data collection for overlooked indicator areas and in countries with low levels of data availability. The application of this indicator framework and resulting data availability analysis could be conducted beyond ASEAN to enable cross-regional benchmarking of health equity.

**Supplementary Information:**

The online version contains supplementary material available at 10.1186/s12939-023-02059-2.

## Background

### Defining and conceptualising health equity

Health inequity is gaining traction in health systems research, and ongoing health disparity around the world has been highlighted as one of the most severe public health threats of the century [5]. The unequal impact of COVID-19 and its mitigation strategies have further highlighted health equity as an issue of major concern, especially with regard to vulnerable populations and their existing health disparities [6–8]. Health equity research must move beyond theoretical discussions and prioritise the *actual* closing of health equity gaps within and among countries [9].

There have been numerous attempts to define health equity. International Organisations have offered broad definitions of the concept. The World Health Organization (WHO) defines health equity as the “absence of **unfair, avoidable or remediable differences** among groups of people, whether those groups are defined socially, economically, demographically, or geographically or by other dimensions of inequality (e.g. sex, gender, ethnicity, disability, or sexual orientation)” [10]. The WHO Commission on Social Determinants of Health similarly defines health inequity as “where systematic differences in health are judged to be **avoidable** by reasonable action” [11]. The Robert Wood Johnson Foundation (RWJF) defines health equity as “**removing obstacles to health** such as poverty, discrimination, and their consequences, including powerlessness and lack of access to good jobs with fair pay, quality education and housing, safe environments, and health care so that everyone has a fair and just opportunity to be as healthy as possible.”[12] These definitions assume that disparities in health outcomes are not unavoidable, but rather emerge from long-standing inequalities in socioeconomic conditions and other processes that underpin health [13].

Efforts have also been made to distinguish the concepts of health equity and health *equality*. According to Braveman, “pursuing health equity means striving for the highest possible standard of health for all people and giving special attention to the needs of those at greatest risk of poor health, based on social conditions.”[14] This does not necessarily entail the equal distribution of health resources. While health inequality refers to mere differences in health within a population, health equity is a function of systemic inequalities between groups [15]. For example - that young adults are generally healthier than older adults is an inevitable inequality; that some racial minorities are more likely to die in childbirth is an example of inequity. Health inequity is therefore the moral and ethical dimension of health inequality [15, 16]

### Measuring health equity in the context of health system development

Achieving health equity is one of the ultimate aims of health systems [17, 18]. While there is sometimes discussion of a health system equity-efficiency tradeoff - whereby providing equitable access to health services diminishes their functional efficiency (e.g. potentially increasing waiting times) - Reidpath et al. argue that achieving health equity should instead be interpreted as *realising* the efficiency of a health system [16]. An equitable distribution of health resources can also help a country achieve good health system performance metrics. According to a review by Rohova, health equity is included as a key dimension in most conceptual frameworks measuring health system performance, covering elements such as equitable access to health, financial protection, health outcomes and quality of health services [19]. Health equity is therefore both an *aim* of health systems and an *element* of good health system performance.

As health equity is ultimately a normative concept, it cannot be measured directly [20]. What is considered ‘fair’ may vary by social context and there may be multiple ways to operationalise equity. A common approach to measuring health equity has been to measure the *lack of inequity*. Braveman argues that, since health equity and health disparity are intertwined, measuring disparities can determine a health system’s progress toward health equity [14]. Monitoring health equity, however, should go beyond measuring health outcome disparities. In line with Dahlgren and Whitehead’s ‘social model of health’, socio-economic and environmental indicators are equally relevant in measuring health equity [21, 22]. Jensen et al. argue for health equity analyses to consider broad structural, political, social and economic drivers of health and the socio-political mechanisms that underpin health system development [23]. Similarly, Amartya Sen argues that “health equity cannot be concerned only with health, seen in isolation. Rather it must come to grips with the larger issue of fairness and justice in social arrangements, including economic allocations, paying appropriate attention to the role of health in human life and freedom” [24].

Various measurement frameworks have been proposed to conceptualise health equity in relation to health system development [16, 23, 25, 26]. Asada proposed a three-step strategy:[16] (i) selecting a suitable definition of health equity (e.g. defining a minimally adequate level of health and understanding whether each individual in a population satisfies this); (ii) selecting a measurement strategy (i.e. selecting the aspects of health equity to measure, and the units of time and analysis to employ); and (iii) quantifying information on health inequity using measures such as the concentration index or Gini coefficient. Anderson developed a roadmap comprising four broad actions:[26] prioritising specific areas in which health disparities are pronounced; implementing evidence-based interventions to reduce these; developing health equity performance measures; and incentivising the reduction of health disparities through new payment models. These frameworks, while successful in conceptualising health equity, do not provide a comprehensive and practical list of indicators to be used to benchmark health equity across health systems.

### Challenges in collecting data on health equity

Health equity poses not only measurement challenges but also challenges in data collection. Collecting data on health equity indicators is essential to setting priorities according to apparent equity gaps and formulating policies to close these gaps [21]. These indicators should cover healthcare access, financial protection, health outcomes and healthcare quality. Data should also be appropriately disaggregated to differentiate between unique subgroups (e.g. degrees of rurality; sub-groups within an ethnic group) and to characterise environmental and structural influences on health [27]. This task is particularly challenging for under-resourced areas and developing health systems where, paradoxically, the need to pay attention to equity in health provision may be stronger than in some mature health systems.

Ideally, data on health equity must be of high quality and must be routinely collected to reflect updated trends [27]. Various international-level databases tracking health equity are available, including the WHO Health Equity Assessment Toolkit and the World Bank Health Equity and Financial Protection Indicators [28, 29]. It will be equally important to track data on health equity at the sub-national and sub-population levels. Data should also be collected on different aspects of health equity: individual and contextual data, such as on social determinants of health or neighbourhood conditions, should be coupled with a consideration of structural determinants such as policies, governance and economics [27]. Each of these levels involves separate challenges in data collection and analysis. Data linkage could facilitate health equity measurements – for example, linking National Census data with disease surveillance data to uncover disparities in pneumococcal disease according to neighbourhood poverty [30]. Yet, this type of data linkage requires a high capacity for demographic surveillance, disease surveillance and information systems. Areas lacking routine data collection, which is often caused by resource constraints, will find themselves at a disadvantage when seeking to monitor health inequities.

### Knowledge and research gaps

Much of the research on health equity and health systems are confined to the conceptual level [16, 23, 25, 26]. Existing literature proposes strategies to operationalise health equity, yet falls short of identifying a comprehensive set of indicators to measure health equity. We believe that identifying a core set of health equity indicators is necessary for application across health systems. These indicators should be collected at the national and sub-national levels to facilitate the development of a health equity index; this index, in turn, could be used to compare how different health systems uphold distributive justice in health. While not all indicators relevant to health equity may be universally reported due to data challenges, we believe that efforts are warranted to propose a set of broad-based indicators that can be routinely collected without much resource expense.

Several empirical studies have been conducted using health equity and/or health system performance indicators. One strand of literature focuses on macro indicators to examine health system performance, but these tend to have little emphasis on health equity [31, 32]. The WHO primary health care measurement framework considers health equity from a systems-level perspective, yet focuses on primary healthcare rather than all levels of service provision [33]. One recent study by Pressman et al. focuses on the development of a health equity index but uses only a disease-specific approach [34]. In country-level studies, the assessment of health equity is generally domain-specific. One Canadian study proposes hospital-level equity indicators as benchmarks for the performance of a local health system,[35] while Indonesian studies have examined catastrophic health payments [36] and quality measurements [37] to evaluate the equity and performance of the health system. Dover and Belon have proposed a comprehensive health equity measurement framework, synthesising pre-existing frameworks and identifying causal relationships between components of health equity such as social circumstances and health-related behaviour [25]. However, while components are listed and examples of indicators are provided to illustrate each component, no full list of indicators is present.

### Aims

There is a need to propose a comprehensive set of indicators to help governments benchmark health equity for health system development. These indicators should be largely objective, intuitive and accessible for most countries. This approach to measuring health equity can offer a standard point of comparison, allowing countries to measure their health equity performance relative to their peers and identify which countries need more support in their data collection efforts. This set of indicators can also be used as a basis to construct a health equity index, which would allow the tracking of countries’ progress toward health equity over time.

Our paper has two aims. First, we aim to develop a conceptual framework by mapping out a core set of indicators to measure health equity and health system development, with a focus on synthesising related health system frameworks and identifying indicators relevant to the ASEAN region. The indicators included in our framework are intended to be practical, usable by most countries and applicable at the international, national and sub-national levels. Second, we aim to understand the current data availability and data challenges involved in measuring health equity for countries in Southeast Asia.

To achieve these aims, we pose the following questions: (i) What are the core indicators needed to construct a health equity and health system development assessment tool? (ii) What is the extent of data availability and what are the data challenges involved?

## Methods

### Comprehensive literature review to construct a health equity framework

To answer the first research question, a comprehensive literature review was undertaken to map out a set of indicators to collect when measuring health equity at the health system level. Relevant studies were identified from major public health academic databases (PubMed, Medline and Scopus). This was followed by a hand search of all publications in the International Journal of Equity in Health from the inception of the journal until February 2022. We also conducted a purposive search of grey literature, such as policy documents and reports from multilateral and non-governmental organisations. Finally, we conducted a snowballing search to identify relevant literature from references in existing studies.

Relevant evidence from January 1991 to February 2022 was identified through two inclusion criteria and three exclusion criteria. The inclusion criteria were: (i) review studies (systematic reviews, scoping reviews or literature reviews identified from the academic database search and reports identified from the grey literature search) published in the English language; (ii) studies examining measurements, benchmarking systems, indicators and metrics for health equity from a health system perspective focusing on the general population. The exclusion criteria were: (i) studies published in languages other than English and prior to 1991; (ii) studies examining measurements, benchmarking systems, indicators and metrics for health equity in a disease-specific or organisation-specific context; (iii) studies examining measurements, benchmarking systems, indicators and metrics for health equity for targeted populations only.

All identified literature was actively screened according to the inclusion criteria by the first, second and fourth authors. Less than 10% of discrepancy was identified among the screening authors, and all discrepancies were resolved through a discussion among all authors.

Relevant data from papers was extracted and a thematic synthesis approach was applied for data analysis (Thomas and Harden 2008). The indicators used in previous literature to measure health equity (e.g. socioeconomic determinants, health financing indicators, rurality etc.) were coded into descriptive themes and used to derive a final set of analytical themes. This process was guided by previous literature on the construction of health equity frameworks and by consultations with research partners in Thailand, Vietnam and Cambodia.

Our final analytical themes formed the domains of our health equity conceptual framework. The specific indicators identified through our comprehensive literature search form the trace indicators under each domain of our conceptual framework.

### Trace indicator data collection to identify data availability and gaps

Following the development of our conceptual framework, we turned our attention to the second research question of the paper: to understand the current data availability for our selected health equity indicators in ASEAN. ASEAN was chosen as there is a lack of health equity research within the region that is sufficiently encompassing and insightful to highlight existing health systems gaps to inform health policy decisions. Moreover, as ASEAN comprises lower-middle-income, upper-middle-income and high-income countries, this enabled us to assess the availability of indicator data across varying levels of development. This review can offer insights into the progress of ASEAN health systems as well as generalisable recommendations for health equity data collection in other regions.

Between September and October 2022, we conducted an active data collection exercise to identify gaps for our 196 framework indicators. We utilised publicly-available databases for this exercise, with the WHO Global Health Observatory and WHO Health Equity Assessment Toolkit as our primary sources. For indicators with no WHO data available, we consulted other international data sources such as the Sustainable Development Report, World Bank Data and Our World in Data directory. For indicators with no data available in international repositories, we collected national-level data from each country’s Ministry of Health website or academic publications.

The latest available data for each indicator was collected in a spreadsheet, and the reference year and data source were noted. When indicator data from the same year was found across multiple platforms, the data from the WHO was prioritised. Indicators with no available data were flagged to highlight gaps in data availability and to inform future iterations of our proposed indicator list.

During data collection, we observed that several indicators in the global databases mimic the original indicators in the conceptual framework that were identified from the comprehensive literature review, but may not correspond exactly to the names of the original indicators. These differences were highlighted and discussed in an online meeting among CB, BM and SYT. It was decided to use the indicator titles most commonly found in global databases and to modify our conceptual framework indicator list accordingly.

## Findings

### Proposing a framework to measure health equity in the context of health system development

**Fig. 1:**
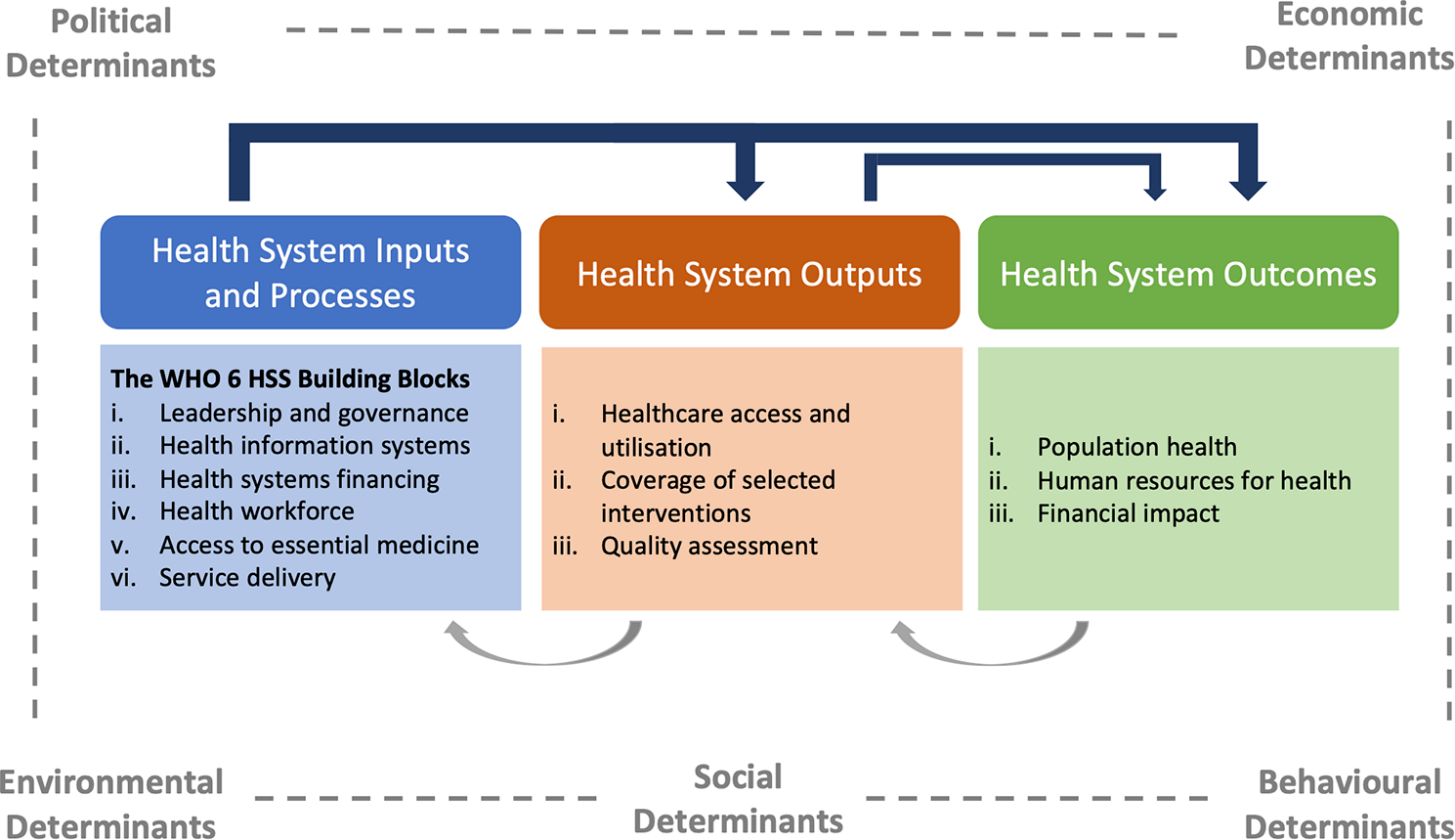
Conceptual framework to measure health equity from a health systems lens

Our proposed conceptual framework (Fig. 1) facilitates the measurement of health equity from a health systems lens. The framework will enable policymakers and practitioners to assess health equity across three stages, or domains: health system inputs and processes, health system outputs and health system outcomes. It also accounts for broader determinants of health equity beyond the immediate realm of the health system. The trace indicators identified under each domain are listed in the Appendix.

#### Health system inputs and processes

This first domain refers to available resources in a health system and its physical and organisational environments. The sub-components that we identified for this domain follow the WHO’s Building Blocks framework for health systems:[38] service delivery, health workforce, health information systems, access to essential medicine, health financing, leadership and governance. The WHO framework continues to inform recent health system research,[39, 40] and its subcomponents are imperative to ensure the equitable distribution of health resources.

#### Health system outputs

This domain represents a health system’s capacity to ensure access to safe, high-quality, effective care. According to a Canadian health system performance measurement framework,[41] access to health services is an intermediate goal which can characterise a health system’s performance in the short- and medium-term. The accessibility of health services shapes patient experiences and care effectiveness, therefore impacting the longer-term outcomes of a health system [41]. Anchoring on this framework, we identified three health system output sub-components: (i) healthcare access and utilisation, (ii) coverage of selected interventions and (iii) quality. To identify relevant trace indicators, we used Levesque’s framework of access to healthcare:[42] ‘access’ is defined as a process spanning the identification of healthcare needs, to seeking healthcare services, reaching the services, and resources, ultimately culminating in various healthcare consequences. We also considered Kruk et al.’s framework to measure health system performance through a focus on “access to high-quality care”, covering to the elements of healthcare access, coverage and (perceived) quality in our health system outputs framework category [43].

#### Health system outcomes

It is crucial to examine how health system inputs and outputs culminate in longer-term health disparities. As our final domain, we take ‘health system outcomes’ to refer to the final goals of a health system. According to the WHO, the primary goal of a health system should be to “improve **population health outcomes** in an equitable way **without overburdening people** with healthcare costs” [44]. We derived three sub-components relevant to this: i) population health outcomes, ii) health financing and iii) human resources for health (HRH). While measures of population health outcomes and health financing directly correspond to the WHO’s definition of a health system, human resources for health are an equally important component identified through our literature review. The availability and quality of HRH is influenced by a health system’s input and output factors (e.g., governance, under- or over-utilisation). In turn, human resources enable the continued provision of care and health system functioning: HRH have been identified as a significant indicator of Universal Health Coverage, and their equitable is a prerequisite to maintaining equity within a health system [45, 46].

Our health equity measurement framework operates sequentially and causally, whereby health system inputs influence health system outputs in the medium-term and health system outcomes in the longer term. Nonetheless, we recognise the complex relationships between all the domains. Health system outcomes, such as population health, also go back to influence health system outputs such as utilisation rates. These outputs can in turn influence health system inputs and processes; for example, coverage levels inform health financing systems by highlighting disease areas that require more funding. Similarly, higher rates of healthcare access and utilisation create a need for more human and non-human resources in service delivery.

#### Contextual factors

Beyond the three core domains of our framework, we identified determinants of health equity that lie beyond the health system. These determinants were prevalent across our literature search due to their relevance to public health and impact at all stages of a health system. They capture large-scale processes such as public policies, cultural values, and environmental conditions in a country, neighbourhood, or population subgroups.

Political determinants (e.g. corruption levels, anti-discrimination laws) can impact the functioning of a health system and a population’s experiences of social equality, social protection and other factors which may influence their health journeys. Social determinants (e.g. educational status) can affect individuals’ health literacy levels, career pathways, access to social capital and other health-relevant experiences. Economic determinants (e.g. presence of a national minimum wage) can affect the sustainability of a health system and individuals’ experiences with income, mental health and financial protection. Behavioural determinants (e.g., prevalence of tobacco use) influence individual health outcomes and population morbidity or health service utilisation rates. Finally, environmental determinants (e.g., neighbourhood crime rates, air quality) can influence individuals’ direct health as well as their health maintenance behaviours such as physical exercise.

### Data availability and gaps of health equity indicators

**Fig. 2:**
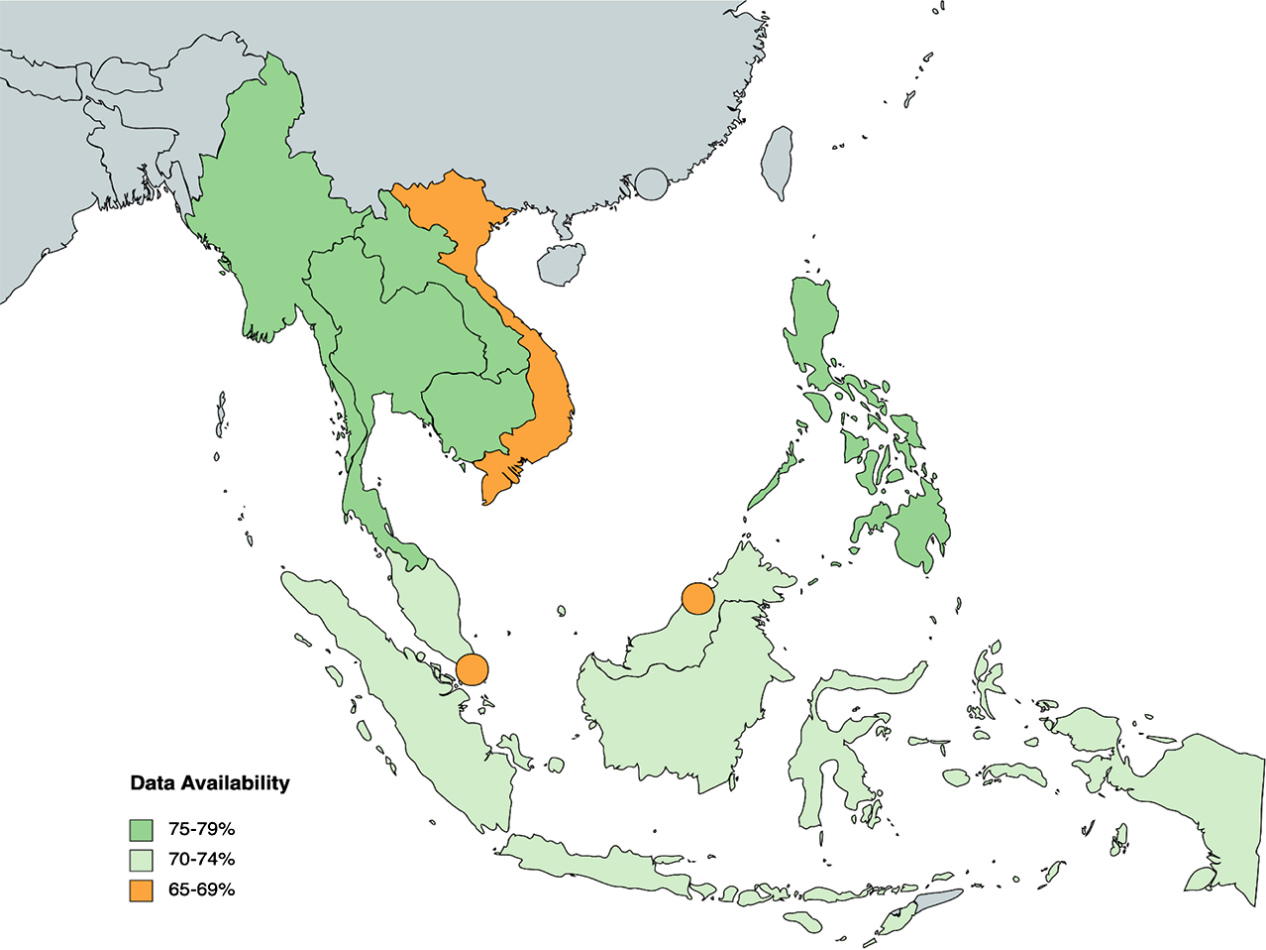
Heat map of overall country data availability for all framework indicators

Our proposed framework identifies a total of **195 indicators** that measure health equity across three health system domains — inputs and processes (64), outputs [36], and outcomes [42] — and contextual factors (53). Data for each indicator was collected between September to October 2022 from publicly available databases on global and national levels. The WHO Global Health Observatory and World Bank served as the primary resources. On average, 73.45% of indicator data is publicly available across the three health systems; 72.97% (1423/1950) of data is publicly available across health system domains and contextual factors. However, only 52.82% (103/195) of all indicators have complete data for 10 ASEAN countries. The Philippines had the highest data availability overall at 77.44% (151/195), while Brunei Darussalam and Vietnam had the lowest data availability at 67.18% (131/195) (see Fig. 2). Period availability of collected data ranged from 2005 to 2022, with 51.95% (1013/1950) of indicator data updated in the past 5 years (since 2018) (see Appendix).

**Fig. 3:**
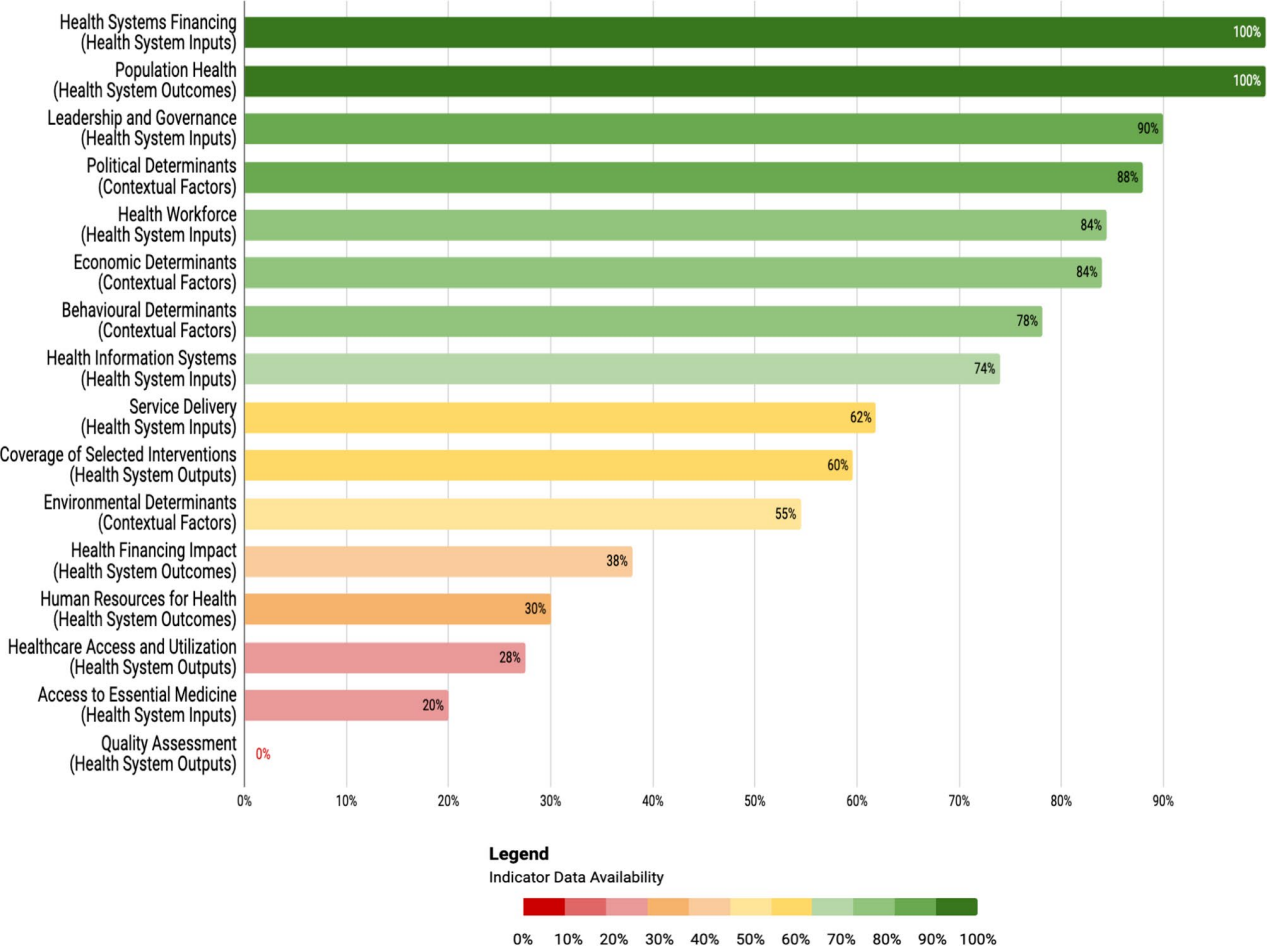
Ranking of all framework sub-components by indicator data availability

### Health system inputs and processes

**Fig. 4:**
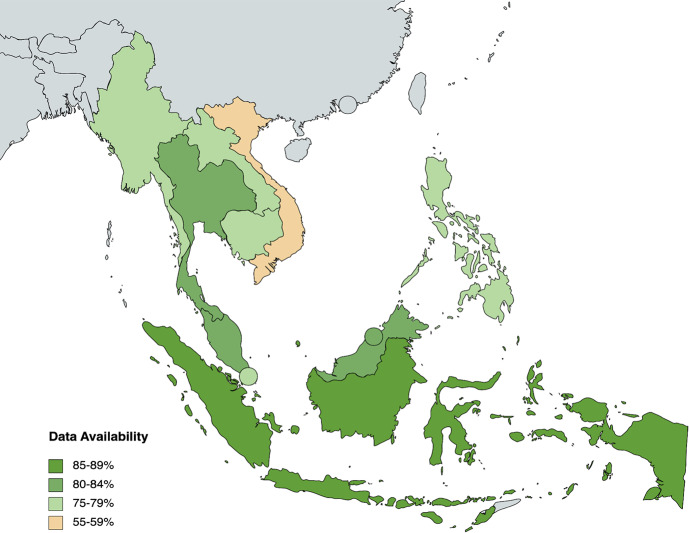
Heat map of country data availability for Health System Inputs and Processes indicators

77.5% (496/640) of data is available for all indicators under Health System Inputs and Processes. 29 out of 64 indicators have complete data across ASEAN countries, while 4 indicators have no data available at all. According to sub-component, 90% of data is available for *Leadership and Governance*, 74% for *Healthcare Information Systems*, 100% for *Health Financing*, 84.42% for *Health Workforce*, 20% for *Access to Essential Medicine*, and 61.88% for *Service Delivery* (see Fig. 3). The significant proportion of data gaps for some categories can be attributed to the lack of data collection for specific indicators related to public, private, secondary and tertiary healthcare. Under *Access to Essential Medicine*, only Indonesia and the Philippines have data available from 2013 — highlighting a lack of screening for this indicator among other countries. Overall, Indonesia has the highest data availability in ASEAN at 84.38% (54/64), while Vietnam has the lowest data availability at 57.81% (37/64) (see Fig. 4).

### Health system outputs

**Fig. 5:**
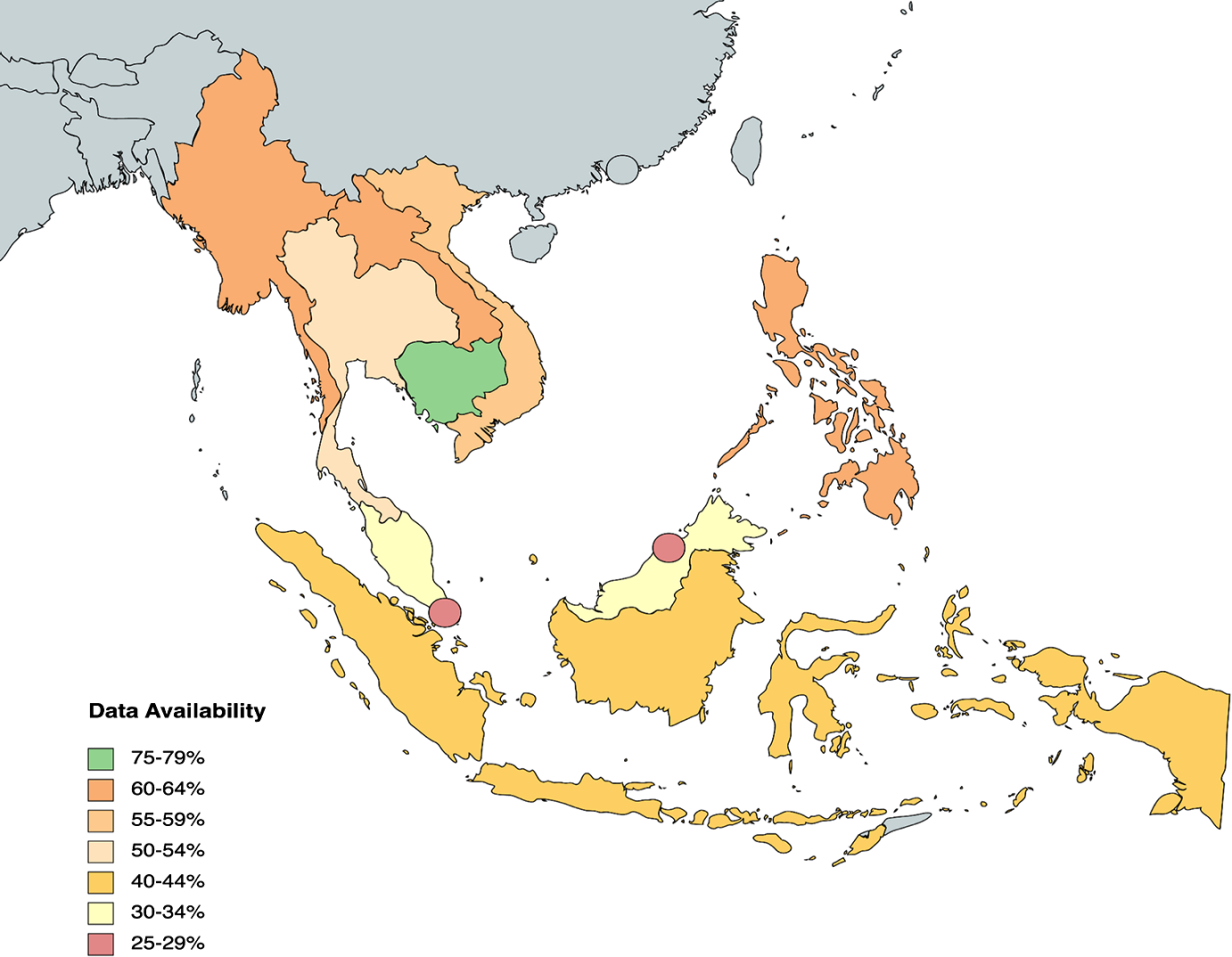
Heat map of country data availability for Health System Outputs indicators

48.89% (180/360) of data is available for all indicators under Health System Outputs. Only 7 out of 36 indicators have complete data across ASEAN countries, while 10 indicators have no data available at all. When considering subcomponents, 27.50% of data is available for *Healthcare Access and Utilisation*, 59.63% for *Coverage of Selected Interventions*, and 0% for *Quality Assessment* (see Fig. 2). As the scope of indicators under the *Healthcare Access and Utilisation* and *Quality Assessment* categories is specified at the public, private, secondary, and tertiary levels of healthcare, data is only available at the national level. On the other hand, data for *Coverage of Selected Interventions* is widely available on international databases. Out of the 10 ASEAN countries, Cambodia has the highest data availability at 69% (25/36), while Brunei Darussalam has the lowest data availability at 25% (9/36) (see Fig. 5).

### Health system outcomes

**Fig. 6:**
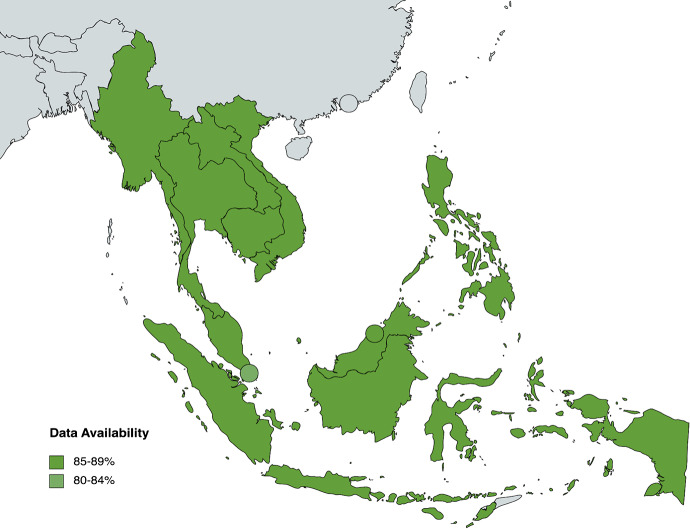
Heat map of country data availability for Health System Outcomes indicators

87.38% (367/420) of data is available for all indicators under Health System Outcomes. 34 out of 42 indicators have complete data for all ASEAN countries, while only 5 indicators have no data available at all. When considering subcomponents, *Population Health* has the highest data availability at 99.43%, followed by *Financial Impact* at 38%, and *Human Resources for Health* at 30% (see Fig. 2). All *Population Health* indicators were collected across 5 international databases, namely the WHO Global Health Observatory, Sustainable Development Report, World Bank, Our World in Data, and World Cancer Research Fund International. While there is no data on the Hepatitis B and Measles Incidence rates per 100,000 population in Singapore, the estimated number of cases for both diseases as of 2022 can be found on the country’s National University Hospital and National Centre for Infectious Diseases websites. Under Human Resources for Health, data on nurse shortage was calculated from the number of nursing personnel under Health System Inputs and Processes using the recommended ratio of 83 nurses per 10,000 population (Health Workforce Status Report 2019). This computation was verified with local news articles from each country on the shortage of nurses in the healthcare system. Overall data availability for this health system is consistent with Singapore averaging the lowest at 83.33% (35/42), followed by Brunei Darussalam at 85.71% (36/42), and the rest of the ASEAN countries averaging 88.10% (37/42) (see Fig. 6).

### Contextual factors

**Fig. 7:**
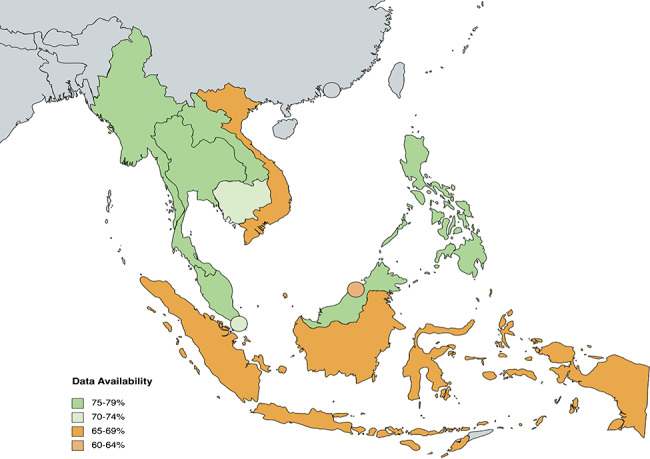
Heat map of country data availability for Contextual Factors indicators

72.45% (384/530) of data is available for all indicators under Contextual Factors. 31 out of 53 indicators have complete data for all ASEAN countries, while 11 indicators have no data available at all — the majority of which fall under Environmental Determinants. When considering subcomponents, *Political Determinants* has the highest data availability at 87.50% (105/120), followed by *Economic Determinants* at 84% (84/100), *Behavioural Determinants* at 78.18% (86/110), and finally, *Environmental Determinants at 54.50% (109/200)* (see Fig. 2). Several contextual factors surrounding health equity within the framework rely on facts — rather than figures — from a country, as evidenced by polar statements such as “state assistance for housing (y/n)” (see Appendix). Among the 10 ASEAN countries, the Philippines has the highest data availability at 75.93% (41/53), and Brunei Darussalam has the lowest at 61.11% (33/53) (see Fig. 7).

## Discussion

### Analysis of results

Our proposed conceptual framework involves a robust set of 196 indicators. These represent all three core domains of the health system (inputs and processes, outputs, and outcomes) and contextual factors surrounding health equity. The health system stages cover a range of assessment areas, from financing to health workforce to population health outcomes. Most of our selected indicators are all-encompassing and applicable to all members of a population, in line with our aim to measure health equity from a health system rather than disease- or subgroup-specific perspective. Nonetheless, some indicators are more specific. For example, indicators on maternal and child health were included within the Health System Outputs and Outcomes categories (e.g. antenatal care coverage (%); birth attended by skilled health staff (%); maternal mortality ratio). While these focus on a specific subpopulation, they also reveal broader truths on the equity of a health system.

It is evident from the heat maps (see Fig. 4 and Appendix) that ASEAN countries must pay more attention to the evaluation of health system outputs. Compared to Health System Inputs and Processes (77.5%) and Outcomes (87.38%), Health System Outputs only have 48.89% of data availability. Across all indicators, the average proportion of data availability sits at 72.60%. A vast majority (27/36, 75%) of Health System Outputs indicators fall below this statistical mean. The data shortcomings of these indicators need to be addressed to understand the relationship between inputs and ultimate outcomes of a health system. Beyond the Health System Outputs component, data availability is below average 19/53 (35.85%) Contextual Factors indicators, 21/64 (32.81%) Health System Inputs and Processes indicators and 2/42 (4.76%) Health System Outcomes indicators. In particular, the Health System Inputs and Processes subcategories of *Access to Essential Medicine* and *Service Delivery* and the Health System Outcomes subcategories of *Human Resources for Health* and *Financial Impact* have data availability averages below the mean and thus require more attention across the region.

### Strengths and limitations

To the best of our knowledge, our paper is among the earliest scholarly works to (1) conceptualise a framework to measure health equity featuring a comprehensive set of indicators, and (2) evaluate the data availability of these indicators in the ASEAN region. Data availability was assessed at both global and national levels, culminating in an extensive review of publicly-available resources. This resulted in a more complete analysis of data availability than would have been produced by utilising a single database. Through the data gaps highlighted, we have identified several overlooked indicator areas that should be prioritised and which ASEAN countries require additional support in collecting data. An important point of inquiry and examination would be the discontinued collection of data for half of the available indicators in the last five years. Applying our proposed framework in regional policy-making could help governments identify lagging areas in health equity and allocate resources to improve equity at each stage of the health system. At the same time, a core set of priority indicators could be identified to ensure that resources for data collection are well-targeted and that continuity across a set of priority indicators is maintained over time.

Expectedly, our paper reveals the lack of consistent data collection practices across ASEAN. This limits the application of our framework in the near future. The data gaps we have identified will have to be addressed by governments to facilitate the development of future iterations of meaningful and actionable health equity assessment frameworks comprising both quantitative and qualitative aspects. In addition, a few chosen indicators are not as relevant to some countries as they are to others. For example, the indicator “use of insecticide-treated bed nets (% of under-5 population)” is only applicable to countries with recent incidences of malaria such as Lao People’s Democratic Republic and Myanmar. While it is imperative to establish a standardised framework for health equity, we need to consider how the extent to which specific indicators that are only relevant to some countries should be incorporated into the framework of health equity assessment without affecting the assessment of other states.

A further limitation involved our inability to consolidate indicator data not readily available in English. Although English is widely used in some Southeast Asian countries - namely Singapore and the Philippines - the majority of national-level health data and communications in other countries are published in the relevant national language. This means that some indicators in non-English-speaking countries may have been marked as ‘unavailable’, even when they may be available in other languages. Nonetheless, our framework aims to enable cross-country benchmarking of health equity and comparative analyses of health systems. We should encourage the consistent data collection of this comprehensive framework of indicators by leveraging a consortium such as the commanding power of the WHO.

### Further gaps

Our research highlights some remaining gaps in the development of a health equity index for health system development. First, there is a need to bridge the gap in data collection at the global and national levels. This requires collaboration among individual country data platforms and regional organisations that maintain global databases (e.g. WHO, World Bank) to determine which indicators should be consistently measured. These indicators must have consistent titles, must be translated into a common language for universal access, and must be measurable at various levels of disaggregation.

Once a common understanding of required indicators to measure health equity is achieved, governments will have to address their data collection gaps. This should include gathering data on more indicators at the national level (including data on contextual factors relevant to health equity), as well as facilitating the collection of more granular and sub-group data segmented according to age, gender, rurality and other demographics. These steps will facilitate the application of a more advanced health equity index based on our framework. Such an index could compare how well countries are performing across different elements of health equity and stages of the health system. This index could also inform on countries’ progress on their health equity performance, highlighting necessary areas of improvement. However, if underlying data for this index is widely unavailable, the ultimate output may not be representative of countries’ *actual* health equity performance or unique areas of priority in terms of health equity and data collection. Therefore, this framework can serve as a foundation for benchmarking health equity, but countries may choose to adapt it according to their own national priorities.

### Next steps for the future development of a health equity index

The next stage of our research will involve developing a health equity index based on our conceptual framework for health equity and its component indicators. We will first produce a pilot index for three ASEAN countries: Thailand, Vietnam and Cambodia. This index will include quantitative factors (i.e. current indicator data; national levels of data availability; remaining data gaps) as well as qualitative factors (i.e. data availability challenges in the three selected countries, as identified through expert stakeholder interviews). The development of an index for Thailand, Vietnam and Cambodia will inform whether a similar process can be conducted across ASEAN and in other regions. Significant data gaps are expected in the initial construction of our index; we plan to revisit our indicator list accordingly to reflect both current and aspirational data collection practices relevant to health equity. This will initially involve identifying a core set of priority indicators for countries to focus on when reporting on levels of health equity across their health systems. Findings from other relevant indices on health equity could help inform the shortlisting of priority indicators for our pilot index. For example, the WHO UHC Service Coverage Index acknowledges the need to collect trace indicators where direct measures of service coverage are unavailable,[47] and an analysis of the Global Burden of Disease Study 2019 found that service coverage for non-communicable diseases in low- and middle-income country lags behind coverage for communicable diseases [48]. These findings will be useful to compare against the current ASEAN landscape and to identify priority areas of missing data. As data on selected core indicators is collected, initial cross-country comparisons can be made and the core indicator list can gradually be expanded to cover more aspects of health equity. In addition, regularly updating the index will enable us to reflect on changes in countries’ health equity performance and efforts to bridge data gaps.

## Conclusion

Narrowing down data availability gaps in the measurement of health equity will enable the identification of areas of health equity to prioritise, and hence be an important prerequisite to closing health gaps among populations. In the ASEAN region, data is more widely available for *Health System Inputs and Processes* indicators relevant to health equity. These include indicators such as health financing and leadership and governance. Nonetheless, significant data gaps remain for *Health System Outputs* and *Outcomes* elements of health equity. While *Input* indicators may be intrinsically easier to measure, these alone cannot explain how health inequity contributes to multiple adverse effects along stages of the health system - from healthcare access, to financing to morbidity and mortality. Increased investment in measuring equity at *all stages of the health system* is therefore warranted. Additionally, there is significant room for improvement in measuring contextual factors relevant to health equity in ASEAN. These include political, social, environmental and other determinants of health. Increasing data availability for these at the national and sub-national level will be challenging, yet critical.

Several papers and reports argue for the need to assess health equity from a multi-dimensional perspective [13, 21, 24]. Our proposed contextual framework for health equity provides a starting point for this and will allow for cross-country evaluations of health equity. Derived from a comprehensive literature search, and refined through country consultations in Thailand, Vietnam and Cambodia, our framework proposes 195 indicators that could be feasibly collected by countries to benchmark their progress toward health equity. In order to achieve this, a policy commitment toward data collection and associated resource investment will be required from ASEAN governments. International organisations such as the WHO can assist countries with lower capacity to improve their data collection practices and to understand the value of multi-dimensional indicators for the improvement of health equity. As data relevant to health equity may fall outside the realm of national Ministries of Health, there should be strong cross-ministerial collaboration to share data and avoid duplicate efforts. Finally, indicators such as those in our proposed framework should be agreed upon by countries to ensure that data is reported in consistent formats, enabling comparative evaluations of countries’ progress toward health equity.

## Electronic supplementary material

Below is the link to the electronic supplementary material.

Supplementary Material 1

Supplementary Material 2

Supplementary Material 3

Supplementary Material 4

## Data Availability

The datasets generated during and/or analysed during the current study are available in the WHO Global Health Observatory repository, https://www.who.int/data/gho^(1)^; Sustainable Development Report repository, https://dashboards.sdgindex.org/map^(2)^; World Bank Open Data repository, https://data.worldbank.org/^(3)^; Our World in Data repository, https://ourworldindata.org/^(4)^; and other online repositories. Further datasets used and/or analysed during the current study are available from the corresponding author on reasonable request.
