# Correction: Measuring health equity in the ASEAN region: conceptual framework and assessment of data availability

**DOI:** 10.1186/s12939-023-02085-0

**Published:** 2024-02-08

**Authors:** Capucine Barcellona, Yzabel Bryanna Mariñas, Si Ying Tan, Gabriel Lee, Khin Chaw Ko, Savina Chham, Chhea Chhorvann, Borwornsom Leerapan, Nam Pham Tien, Jeremy Lim

**Affiliations:** 1 Saw Swee Hock School of Public Health, National University of Singapore, Singapore, Singapore; 2 Yale-NUS College, National University of Singapore, Singapore, Singapore; 3 Independent Consultant, Singapore, Singapore; 4 National Institute of Public Health Cambodia, Phnom Penh, Cambodia; 5 Faculty of Medicine Ramathibodi Hospital, Hanoi University of Public Health, Hanoi, Vietnam; 6 Hanoi University of Public Health, Hanoi, Vietnam

**International Journal for Equity in Health (2023) 22:251**

10.1186/s12939-023-02059-2.

After publication of this article [1], the authors reported that in this article the author name Yzabel Bryanna Mariñas, was incorrectly written as Bryanna Yzabel Mariñas.

The original article [1] has been corrected.
