# Correction: Measuring health equity in the ASEAN region: conceptual framework and assessment of data availability

**DOI:** 10.1186/s12939-024-02276-3

**Published:** 2024-09-29

**Authors:** Capucine Barcellona, Yzabel Bryanna Mariñas, Si Ying Tan, Gabriel Lee, Khin Chaw Ko, Savina Chham, Chhea Chhorvann, Borwornsom Leerapan, Nam Pham Tien, Jeremy Lim

**Affiliations:** 1 Saw Swee Hock School of Public Health, National University of Singapore, Singapore, Singapore; 2 Yale-NUS College, National University of Singapore, Singapore, Singapore; 3 Independent Consultant, Singapore, Singapore; 4 National Institute of Public Health Cambodia, Phnom Penh, Cambodia; 5 Faculty of Medicine Ramathibodi Hospital, Mahidol University, Bangkok, Thailand; 6 Hanoi University of Public Health, Hanoi, Vietnam

**Correction:** ***Int J Equity Health*** **22, 251 (2023)**

10.1186/s12939-023-02059-2

Following publication of the original article [1], the authors reported 1 error that the author Borwornsom Leerapan’s affiliation should be corrected from Faculty of Medicine Ramathibodi Hospital, Hanoi University of Public Health, Hanoi, Vietnam to Faculty of Medicine Ramathibodi Hospital, Mahidol University, Bangkok, Thailand.

The original article [1] has been updated.
