# Supplementary material for: Measuring health equity in the ASEAN region: conceptual framework and assessment of data availability
Source: Int J Equity Health. 2023 Dec 5;22:251. doi: 10.1186/s12939-023-02059-2 (PMC10696689; doi:10.1186/s12939-023-02059-2)
Supplement: Supplementary file 1 — Supplementary Material 1 [file 12939_2023_2059_MOESM1_ESM.pdf]

Health System Output 1: Healthcare Access and Utilization

Country	Brunei Darussalam		Cambodia		Indonesia		Lao PDR		Malaysia		Myanmar		Philippines		Singapore		Thailand		Vietnam		
**Indicator**	**Value**	**Year**	**Value**	**Year**	**Value**	**Year**	**Value**	**Year**	**Value**	**Year**	**Value**	**Year**	**Value**	**Year**	**Value**	**Year**	**Value**	**Year**	**Value**	**Year**	**% Availability**
Utilization of Outpatient Care																					**60%**
Outpatient Visits (per 100,000)	10516	2014	326.5	2014	ND	ND	0.044	2014	ND	ND	108.2	2014	ND	ND	4294	2014	5435	2014	ND	ND	60%
Utilization of Inpatient Care																					**23%**
Utilization of STHC inpatient services (admissions per person per year) (public vs pte facilities)	ND	ND	ND	ND	ND	ND	ND	ND	ND	ND	ND	ND	ND	ND	ND	ND	ND	ND	ND	ND	0%
Inpatient bed-days per person per year (public vs pte) - *length of stay*	5.3	2015	5	2015	4 - 5.4	2017	2	2016	3.11 - 3.17	2010	5	2017	3	2021	4.64	2018	ND	ND	7.2 (province)	2019	90%
Total no. or % inpatient admissions in public secondary and tertiary healthcare (SHTC)	ND	ND	ND	ND	ND	ND	ND	ND	ND	ND	ND	ND	ND	ND	ND	ND	ND	ND	ND	ND	0%
Total no. or % inpatient admissions in private SHTC	ND	ND	ND	ND	ND	ND	ND	ND	ND	ND	ND	ND	ND	ND	ND	ND	ND	ND	ND	ND	0%
Utilization of Dental Care																					**0%**
No. of dental health visits per person per year	ND	ND	ND	ND	ND	ND	ND	ND	ND	ND	ND	ND	ND	ND	ND	ND	ND	ND	ND	ND	0%
																					**27.50%**

Health System Output 2: Coverage of Selected Interventions

Country	Brunei Darussalam		Cambodia		Indonesia		Lao PDR		Malaysia		Myanmar		Philippines		Singapore		Thailand		Vietnam		
**Indicator**	**Value**	**Year**	**Value**	**Year**	**Value**	**Year**	**Value**	**Year**	**Value**	**Year**	**Value**	**Year**	**Value**	**Year**	**Value**	**Year**	**Value**	**Year**	**Value**	**Year**	**% Availability**
Health Insurance Enrollment Rate	ND	ND	ND	ND	ND	ND	ND	ND	ND	ND	ND	ND	ND	ND	ND	ND	ND	ND	ND	ND	0%
Demand for family planning services (%) (for example: % of women of reproductive age (15–49 years) who are married or in- union who have their need for family planning satisfied with modern methods)	ND	ND	63.6	2022	81.4	2022	75.5	2022	57.6	2022	78.9	2022	59.2	2022	77.6	2022	91.4	2022	79.6	2022	90%
Antenatal care coverage (at least 1 visit) (%) (in the two or three years preceding the survey) - 15-19 years	ND	ND	96	2014	ND	ND	75	2017	ND	ND	80	2015	91	2017	ND	ND	98	2019	91	2013	60%
Antenatal care coverage (at least 1 visit) (%) (in the two or three years preceding the survey) - 20-49 years	ND	ND	96	2014	ND	ND	79	2017	ND	ND	83	2015	94	2017	ND	ND	99	2019	96	2013	60%
Antenatal care coverage (at least 1 visit) (%) (in the two or three years preceding the survey) - Rural	ND	ND	96	2014	ND	ND	73	2017	ND	ND	79	2015	93	2017	ND	ND	98	2019	94	2013	60%
Antenatal care coverage (at least 1 visit) (%) (in the two or three years preceding the survey) - Urban	ND	ND	99	2014	ND	ND	93	2017	ND	ND	94	2015	93	2017	ND	ND	99	2019	99	2013	60%
Antenatal care coverage (at least 1 visit) (%) (in the five years preceding the survey) - 15-19 years	ND	ND	95	2014	ND	ND	ND	ND	ND	ND	77	2015	91	2017	ND	ND	ND	ND	ND	ND	30%
Antenatal care coverage (at least 1 visit) (%) (in the five years preceding the survey) - 20-49 years	ND	ND	95	2014	ND	ND	ND	ND	ND	ND	81	2015	94	2017	ND	ND	ND	ND	ND	ND	30%
Antenatal care coverage (at least 1 visit) (%) (in the five years preceding the survey) - Rural	ND	ND	95	2014	ND	ND	ND	ND	ND	ND	76	2015	94	2017	ND	ND	ND	ND	ND	ND	30%
Antenatal care coverage (at least 1 visit) (%) (in the five years preceding the survey) - Urban	ND	ND	99	2014	ND	ND	ND	ND	ND	ND	94	2015	94	2017	ND	ND	ND	ND	ND	ND	30%
Antenatal care coverage (at least 4 visits) (%)	100	2016	75.6	2014	90.6	2017	62.2	2017	97.4	16	58.6	2016	86.5	2017	97.4	2020	90	2019	73.7	2014	100%
Births attended by skilled health staff (% of total)	99.8	2017	89	2014	94.7	2019	64.4	2017	99.6	2017	60.2	2016	84.4	2017	99.5	2018	99.1	2016	93.8	2014	100%
Children under-5 with diarrhea treated with oral rehydration therapy (%)	ND	ND	52	2014	61	2017	61	2017	ND	ND	56	2016	45	2017	ND	ND	71	2016	58	2014	70%
Children aged < 5 years with pneumonia symptoms taken to a health facility (%) - Male	ND	ND	62	2014	76	2017	48	2017	ND	ND	65	2015	70	2017	ND	ND	76	2015	85	2013	70%
Children aged < 5 years with pneumonia symptoms taken to a health facility (%) - Female	ND	ND	76	2014	73	2017	30	2017	ND	ND	49	2015	60	2017	ND	ND	84	2015	76	2013	70%
Immunization, measles (% of children ages 12-23 months)	99	2020	84	2020	76	2020	79	2020	95	2020	91	2020	72	2020	95	2019	96	2020	97	2020	100%
Immunization, DPT (% of children ages 12-23 months)	99	2020	92	2020	77	2020	79	2020	98	2020	84	2020	71	2020	96	2020	97	2020	94	2020	100%
Coverage of mental health services (%)	ND	ND	ND	ND	ND	ND	ND	ND	ND	ND	ND	ND	ND	ND	ND	ND	ND	ND	ND	ND	0%
Treatment coverage of opioid dependence (%)	ND	ND	ND	ND	ND	ND	ND	ND	ND	ND	ND	ND	ND	ND	ND	ND	ND	ND	ND	ND	0%
Tuberculosis Relapse Cases - Extrapulmonary	2	2020	44	2020	491	2020	2	2020	179	2020	490	2020	265	2020	28	2020	592	2020	324	2020	100%
Tuberculosis Relapse Cases - Pulmonary, bacteriologically confirmed	16	2020	266	2020	7554	2020	181	2020	804	2020	5385	2020	10343	2020	55	2020	3441	2020	4972	2020	100%
Tuberculosis treatment (% of incidence TB cases that are detected and successfully treated in a given year)	75	2019	96	2019	83	2019	90	2019	80	2019	88	2019	86	2019	79	2019	85	2019	91	2019	100%
Children with fever receiving antimalarial drugs (% of children under age 5 with fever)	ND	ND	0.3	2014	0.2	2017	1.9	2012	ND	ND	0.8	2016	0.3	2017	ND	ND	ND	ND	1.2	2011	60%
Use of insecticide-treated bed nets (% of under-5 population)	ND	ND	4	2006	3.7	2007	49.8	2017	ND	ND	18.6	2016	ND	ND	ND	ND	ND	ND	94	2011	50%
Adults & children currently receiving ARV - Male	ND	ND	86	2020	28	2020	53	2020	48	2020	ND	ND	43	2020	ND	ND	78	2020	66	2020	70%
Adults & children currently receiving ARV - Female	ND	ND	82	2020	24	2020	56	2020	63	2020	ND	ND	25	2020	ND	ND	79	2020	72	2020	70%
Key populations at higher risk who have received an HIV test in the past 12 months and know their results (PWID / SW / MSM)	ND	ND	ND	ND	ND	ND	ND	ND	ND	ND	ND	ND	ND	ND	ND	ND	ND	ND	ND	ND	0%
																					**59.63%**

Health System Output 3: Quality Assessment

Country	Brunei Darussalam		Cambodia		Indonesia		Lao PDR		Malaysia		Myanmar		Philippines		Singapore		Thailand		Vietnam		
**Indicator**	**Value**	**Year**	**Value**	**Year**	**Value**	**Year**	**Value**	**Year**	**Value**	**Year**	**Value**	**Year**	**Value**	**Year**	**Value**	**Year**	**Value**	**Year**	**Value**	**Year**	**% Availability**
Presence of national survey on citizens' perceptions of service quality (e.g. waiting times, amenities, choice etc.)	ND	ND	ND	ND	ND	ND	ND	ND	ND	ND	ND	ND	ND	ND	ND	ND	ND	ND	ND	ND	0%
Average waiting time – i.e. hip fracture surgery	ND	ND	ND	ND	ND	ND	ND	ND	ND	ND	ND	ND	ND	ND	ND	ND	ND	ND	ND	ND	0%
Average waiting time – i.e. elective surgery	ND	ND	ND	ND	ND	ND	ND	ND	ND	ND	ND	ND	ND	ND	ND	ND	ND	ND	ND	ND	0%
																					**0%**

**% Availability (36 indicators)**	**25.00%**		**69.44%**		**44.44%**		**58.33%**		**30.56%**		**63.89%**		**63.89%**		**27.78%**		**50.00%**		**55.56%**		**48.89%**
	9	36	25	36	16	36	21	36	11	36	23	36	23	36	10	36	18	36	20	36	
