# Supplementary material for: Measuring health equity in the ASEAN region: conceptual framework and assessment of data availability
Source: Int J Equity Health. 2023 Dec 5;22:251. doi: 10.1186/s12939-023-02059-2 (PMC10696689; doi:10.1186/s12939-023-02059-2)
Supplement: Supplementary file 2 — Supplementary Material 2 [file 12939_2023_2059_MOESM2_ESM.pdf]

Health System Outcome 1: Population Health

Country	Brunei Darussalam		Cambodia		Indonesia		Lao PDR		Malaysia		Myanmar		Philippines		Singapore		Thailand		Vietnam		
**Indicator**	**Value**	**Year**	**Value**	**Year**	**Value**	**Year**	**Value**	**Year**	**Value**	**Year**	**Value**	**Year**	**Value**	**Year**	**Value**	**Year**	**Value**	**Year**	**Value**	**Year**	**% Availability**
Life Expectancy																					**60%**
Life Expectancy at Birth (Years) - Male	75	2020	68	2020	70	2020	66	2020	74	2020	64	2020	67	2020	82	2020	74	2020	71	2020	100%
Life Expectancy at Birth (Years) - Female	77	2020	72	2020	74	2020	70	2020	78	2020	70	2020	76	2020	86	2020	81	2020	80	2020	100%
Life Expectancy at Age 60 Years (Years) - Male	20.2	2017	16.6	2017	16.8	2017	16	2017	19.8	2017	15.4	2017	16.7	2017	23.5	2017	20.8	2017	19.4	2017	100%
Life Expectancy at Age 60 Years (Years) - Female	21.1	2017	18.3	2017	19.7	2017	17.8	2017	21.4	2017	18	2017	22	2017	27	2017	24.4	2017	23.9	2017	100%
Healthy Life Expectancy (HALE) at Birth (Years) - Male	65	2019	60	2019	62	2019	59	2019	65	2019	59	2019	60	2019	72	2019	66	2019	62	2019	100%
Healthy Life Expectancy (HALE) at Birth (Years) - Female	6600%	2019	63	2019	64	2019	62	2019	67	2019	63	2019	64	2019	75	2019	71	2019	6800%	2019	100%
Healthy Life Expectancy (HALE) at Age 60 Years (Years) - Male	1432%	2019	11.97	2019	12.66	2019	12.52	2019	14.02	2019	12.38	2019	12.12	2019	18.81	2019	16.99	2019	12.91	2019	100%
Healthy Life Expectancy (HALE) at Age 60 Years (Years) - Female	14.67	2019	14.09	2019	14.04	2019	14.04	2019	15.26	2019	14.58	2019	14.62	2019	21.05	2019	18.8	2019	16.4	2019	100%
Mortality																					**100%**
Mortality per 1,000 Live Births - Neonatal	6	2020	13	2020	12	2020	22	2020	5	2020	22	2020	13	2020	1	2020	5	2020	10	2020	100%
Mortality per 1,000 Live Births - Infant	10	2020	22	2020	20	2020	35	2020	7	2020	35	2020	21	2020	2	2020	7	2020	17	2020	100%
Mortality per 1,000 Live Births - Under-5	12	2020	26	2020	23	2020	44	2020	9	2020	44	2020	26	2020	2	2020	9	2020	21	2020	100%
Maternal Mortality Ratio (per 100,000 live births)	31	2017	160	2017	177	2017	185	2017	29	2017	250	2017	121	2017	8	2017	37	2017	43	2017	100%
Probability of Dying Between Age 30-70 from any COV, Cancer, Diabetes, Chronic Respiratory Diseases (*ncdmr*) - Male	20	2019	27	2019	28	2019	31	2019	22	2019	31	2019	30	2019	12	2019	17	2019	29	2019	100%
Probability of Dying Between Age 30-70 from any COV, Cancer, Diabetes, Chronic Respiratory Diseases (*ncdmr*) - Female	17	2019	19	2019	21	2019	23	2019	15	2019	20	2019	19	2019	7	2019	11	2019	14	2019	100%
Deaths Attributed to Road Traffic Injuries (per 100,000 population)	8	2019	20	2019	11	2019	18	2019	23	2019	20	2019	12	2019	2	2019	32	2019	31	2019	100%
Deaths Attributed to HH and Ambient Air Pollution (per 100,000 population) - Male	15	2016	164	2016	134	2016	196	2016	56	2016	188	2016	225	2016	33	2016	79	2016	95	2016	100%
Deaths Attributed to HH and Ambient Air Pollution (per 100,000 population) - Female	11	2016	140	2016	94	2016	182	2016	39	2016	133	2016	151	2016	19	2016	47	2016	43	2016	100%
Deaths Attributed to Unsafe Water, Unsafe Sanitation, and Lack of Hygiene (per 100,000 population) - Male	0	2016	7	2016	6	2016	11	2016	0	2016	13	2016	5	2016	0	2016	4	2016	2	2016	100%
Deaths Attributed to Unsafe Water, Unsafe Sanitation, and Lack of Hygiene (per 100,000 population) - Female	0	2016	6	2016	8	2016	11	2016	0	2016	12	2016	4	2016	0	2016	3	2016	1	2016	100%
Suicide Mortality (per 100,000 population) - Male	4	2019	7	2019	4	2019	8	2019	9	2019	5	2019	3	2019	15	2019	15	2019	10	2019	100%
Suicide Mortality (per 100,000 population) - Female	1	2019	3	2019	1	2019	3	2019	2	2019	1	2019	1	2019	7	2019	3	2019	5	2019	100%
Morbidity																					**99%**
Cancer Incidence (per 100,000 population)	971	2022	18120	2022	389768	2022	9025	2022	48160	2022	72907	2022	152803	2022	23260	2022	187677	2022	181333	2022	100%
Cancer Incidence (per 100,000 population) - ASR/100,000	219.5	2022	133.3	2022	138.4	2022	163.7	2022	142.5	2022	133.6	2022	161	2022	229.8	2022	161.7	2022	158.6	2022	100%
TB Incidence (per 100,000 population) - Male	101	2020	331	2020	345	2020	192	2020	108	2020	400	2020	754	2020	52	2020	206	2020	253	2020	100%
TB Incidence (per 100,000 population) - Female	67	2020	222	2020	257	2020	105	2020	70	2020	220	2020	323	2020	39	2020	98	2020	101	2020	100%
Hepatitis B incidence (per 100,000 population)	464.4	2019	1848.29	2019	1144.05	2019	2009.82	2019	544.84	2019	636.23	2019	1685.81	2019	ND	ND	1378.93	2019	1972.64	2019	90%
Malaria (per 1,000 population at risk)	0	2020	5.8	2020	2.9	2020	1.5	2020	0	2020	2.5	2020	0.7	2020	0	2020	0.2	2020	0	2020	100%
Measles (per 100,000 population)	0	2021	5	2021	394	2021	2	2021	128	2021	8	2021	206	2021	ND	ND	66	2021	162	2021	90%
HIV incidence (new infections per 1,000 population)	1	2020	0.1	2020	0.1	2020	0.1	2020	0.2	2020	1	2020	0.2	2020	0	2020	0.1	2020	0.1	2020	100%
Prevalence of Mental Disorders (Age-Standardized) (%)	10.36	2019	12.36	2019	10.68	2019	12.04	2019	12.23	2019	10.89	2019	11.94	2019	ND	ND	12.04	2019	9.52	2019	100%
Diabetes prevalence (% of population ages 20 to 79)	11.1	2021	7.3	2021	10.6	2021	6.2	2021	19	2021	7.1	2021	7.1	2021	11.6	2021	9.7	2021	6.1	2021	100%
Incidence of Cardiovascular Diseases (High Blood Pressure, Hyperlipidemia, Hypertension) (per 100,000 population) - Male	47	2019	26	2019	36	2019	26	2019	41	2019	35	2019	35	2019	35	2019	29	2018	33	2019	100%
Incidence of Cardiovascular Diseases (High Blood Pressure, Hyperlipidemia, Hypertension) (per 100,000 population) - Female	46	2019	25	2019	45	2019	31	2019	41	2019	40	2019	33	2019	27	2019	29	2018	26	2019	100%
Tobacco Control (Age-Standardized Prevalence of Adults >= 15 Years Smoking Tobacco in Last 30 Days - Male	30	2020	36	2020	71	2020	53	2020	44	2020	69	2020	39	2020	28	2020	41	2020	47	2020	100%
Tobacco Control (Age-Standardized Prevalence of Adults >= 15 Years Smoking Tobacco in Last 30 Days - Female	2	2020	6	2020	4	2020	10	2020	1	2020	20	2020	7	2020	5	2020	3	2020	2	2020	100%
																					**99.43%**

Health System Outcome 2: Human Resources for Health

Country	Brunei Darussalam		Cambodia		Indonesia		Lao PDR		Malaysia		Myanmar		Philippines		Singapore		Thailand		Vietnam		
**Indicator**	**Value**	**Year**	**Value**	**Year**	**Value**	**Year**	**Value**	**Year**	**Value**	**Year**	**Value**	**Year**	**Value**	**Year**	**Value**	**Year**	**Value**	**Year**	**Value**	**Year**	**% Availability**
The extent of international migration of health workforce	ND	ND	ND	ND	ND	ND	ND	ND	ND	ND	ND	ND	ND	ND	ND	ND	ND	ND	ND	ND	0%
Physician shortage	ND	ND	ND	ND	ND	ND	ND	ND	ND	ND	ND	ND	ND	ND	ND	ND	ND	ND	ND	ND	0%
Nurse Shortage (recommended ratio: 83/10,000)	Yes	2022	Yes	2022	Yes	2022	Yes	2020	Yes	2022	Yes	2021	Yes	2022	Yes	2020	Yes	2022	Yes	2022	100%
																					**33.30%**

Health System Outcome 3: Financial Impact

Country	Brunei Darussalam		Cambodia		Indonesia		Lao PDR		Malaysia		Myanmar		Philippines		Singapore		Thailand		Vietnam		
**Indicator**	**Value**	**Year**	**Value**	**Year**	**Value**	**Year**	**Value**	**Year**	**Value**	**Year**	**Value**	**Year**	**Value**	**Year**	**Value**	**Year**	**Value**	**Year**	**Value**	**Year**	**% Availability**
Population with household expenditures on health greater than 25% of total household expenditure or income (SDG indicator 3.8.2) (%)	ND	ND	4.92	2019	0.87	2017	0.26	2007	0.13	2019	3.47	2017	1.41	2015	1.47	2013	0.27	2019	1.73	2020	90%
Extent of catastrophic health expenditure: Incidence of catastrophic expenditure at 25% of hh consumption or income	ND	ND	ND	ND	ND	ND	ND	ND	ND	ND	ND	ND	ND	ND	ND	ND	ND	ND	ND	ND	0%
Extent of catastrophic health expenditure: Incidence of impoverishment due to OOP expenditure	ND	ND	ND	ND	ND	ND	ND	ND	ND	ND	ND	ND	ND	ND	ND	ND	ND	ND	ND	ND	0%
																					**30.00%**

	Brunei Darussalam		Cambodia		Indonesia		Lao PDR		Malaysia		Myanmar		Philippines		Singapore		Thailand		Vietnam		
**% Availability (42 indicators)**	**85.71%**		**88.10%**		**88.10%**		**88.10%**		**88.10%**		**88.10%**		**88.10%**		**83.33%**		**88.10%**		**88.10%**		**87.38%**
	36	42	37	42	37	42	37	42	37	42	37	42	37	42	35	42	37	42	37	42	
