# Supplementary material for: Measuring health equity in the ASEAN region: conceptual framework and assessment of data availability
Source: Int J Equity Health. 2023 Dec 5;22:251. doi: 10.1186/s12939-023-02059-2 (PMC10696689; doi:10.1186/s12939-023-02059-2)
Supplement: Supplementary file 3 — Supplementary Material 3 [file 12939_2023_2059_MOESM3_ESM.pdf]

Contextual Factors: Behavioural Determinants

Country	Brunei Darussalam		Cambodia		Indonesia		Lao PDR		Malaysia		Myanmar		Philippines		Singapore		Thailand		Vietnam		
**Indicator**	**Value**	**Year**	**Value**	**Year**	**Value**	**Year**	**Value**	**Year**	**Value**	**Year**	**Value**	**Year**	**Value**	**Year**	**Value**	**Year**	**Value**	**Year**	**Value**	**Year**	**% Availability**
Prevalence of tobacco use (% adults)	21.5	2018	21.8	2018	33.8	2018	32.1	2018	22.8	2018	54.4	2018	23.8	2018	ND	ND	20.7	2018	24.9	2018	90%
Prevalence of tobacco use (% adolescents)	9.2	2020	2.4	2020	ND	ND	11.1	2020	20.9	2020	13.6	2020	16	2020	ND	ND	15	2020	ND	ND	70%
Drug use (% adults)	ND	ND	ND	ND	ND	ND	ND	ND	ND	ND	ND	ND	ND	ND	ND	ND	ND	ND	ND	ND	0%
Opioid-related hospitalisations	ND	ND	ND	ND	ND	ND	ND	ND	ND	ND	ND	ND	ND	ND	ND	ND	ND	ND	ND	ND	0%
Alcohol, total per capita (15+) consumption<br>(in litres of pure alcohol) (SDG Indicator 3.5.2)	0.46<br>[0.15 - 0.97]	2019	7.84<br>[6.16 - 9.7]	2019	0.22<br>[0.091 - 0.5]	2019	12.08<br>[10.11 - 14.06]	2019	0.93<br>[0.59 - 1.42]	2019	2.14<br>[1.75 - 2.65]	2019	7.02<br>[5.61 - 8.42]	2019	2<br>[1.61 - 2.54]	2019	8.5<br>[7.04 - 10.19]	2019	7.93<br>[6.08 - 9.68]	2019	100%
Alcohol, consumers past 12 months (%)	3330%	2016	30.9	2016	22.7	2016	40	2016	29.6	2016	27.1	2016	33.1	2016	70	2016	40.9	2016	9940%	2016	100%
15-19 years old, current drinkers (%)	24.5<br>[20 - 29.4]	2016	19.2<br>[17.5 - 21]	2016	13.1<br>[11.7 - 14.6]	2016	26.4<br>[24.3 - 28.8]	2016	17.5<br>[16.2 - 20]	2016	16.2<br>[14.8 - 18]	2016	20.8<br>[19 - 22.7]	2016	57.2<br>[50.3 - 63.2]	2016	27.3<br>[25.2 - 29.7]	2016	23.4<br>[21.4 - 25.5]	2016	100%
Prevalence of insufficient physical activity among<br>school going adolescents aged 11-17 years	87.1<br>[84.27 - 89.51]	2016	91.58<br>[89.62 - 93.2]	2016	86.38<br>[83.58 - 88.77]	2016	84.43<br>[81.46 - 87.01]	2016	86.17<br>[83.15 - 88.73]	2016	86.81<br>[84.11 - 89.12]	2016	93.41<br>[91.94 - 94.63]	2016	76.27<br>[64.99 - 84.82]	2016	77.47<br>[73.61 - 80.92]	2016	86.32<br>[83.54 - 88.69]	2016	100%
Prevalence of insufficient physical activity among<br>adults aged 18+ years (crude estimate) (%)	26.48<br>[17.84 - 37.29]	2016	9.82<br>[6.42 - 14.72]	2016	21.54<br>[15.15 - 29.66]	2016	15.03<br>[11.22 - 18.84]	2016	37.73<br>[28.75 - 47.59]	2016	10.37<br>[7.26 - 14.58]	2016	38.21<br>[29.98 - 47.06]	2016	37.89<br>[22.74 - 55.77]	2016	25.37<br>[18.8 - 33.24]	2016	25.05<br>[18.66 - 32.67]	2016	100%
Prevalence of obesity among adults,<br>BMI >= 30 (age-standardized estimate) (%)	14.1<br>[10.5 - 18.3]	2016	3.9<br>[2.5 - 5.6]	2016	6.9<br>[5 - 9]	2016	5.3<br>[3.5 - 7.6]	2016	15.6<br>[12.5 - 19]	2016	5.8<br>[4.1 - 7.9]	2016	6.4<br>[4.6 - 8.7]	2016	6.1<br>[4.2 - 8.3]	2016	10<br>[7.4 - 13.1]	2016	2.1<br>[1.4 - 3.1]	2016	100%
Prevalence of obesity among children and adolescents, BMI > +2<br>standard deviations above the median (crude estimate) (%)	12.4<br>[7.4 - 18.6]	2016	2.5<br>[0.6 - 6.4]	2016	4.9<br>[3 - 7.2]	2016	3.6<br>[1.1 - 7.9]	2016	11.3<br>[8 - 15.2]	2016	2.9<br>[1.1 - 6.1]	2016	3.4<br>[1.7 - 5.9]	2016	5.7<br>[1.8 - 11.8]	2016	9.6<br>[5.4 - 14.8]	2016	1.6<br>[0.6 - 3.1]	2016	100%
																					**78.18%**

Contextual Factors: Political Determinants

Country	Brunei Darussalam		Cambodia		Indonesia		Lao PDR		Malaysia		Myanmar		Philippines		Singapore		Thailand		Vietnam		
**Indicator**	**Value**	**Year**	**Value**	**Year**	**Value**	**Year**	**Value**	**Year**	**Value**	**Year**	**Value**	**Year**	**Value**	**Year**	**Value**	**Year**	**Value**	**Year**	**Value**	**Year**	**% Availability**
Corruption Perception Index Score	60/100	2020	23/100	2021	38/100	2021	30/100	2021	48/100	2021	28/100	2021	33/100	2021	85/100	2021	35/100	2021	39/100	2021	100%
(Perceived Democratic Deficit) Democrcacy Index Score	ND	ND	ND	ND	ND	ND	ND	ND	23	2022	ND	ND	12	2022	17	2022	40	2022	11	2022	50%
Social democratic welfare state (Y/N)	No	2022	No	2022	No	2022	No	2022	Yes?	2022	Yes?	2022	Yes	2022	No	2022	No	2022	Yes	2022	100%
Low Institute Asia Power Index	15.5	2022	18.3	2022	37.6	2022	19	2022	11.5	2022	22.6	2022	24	2022	23.7	2022	21.3	2022	5.4	2022	100%
National or subnational policy addressing the<br>reduction of health inequities established and documented	Yes	2022	Yes	2022	No	2022	Yes	2022	Yes	2022	No?	2022	Yes	2022	Yes	2022	Yes?	2022	Yes?	2022	100%
Gender Parity index (female or male) for the percentage of the<br>population covered by social protection floors or systems (%)	67.80%	2021	68.40%	2021	68.80%	2021	75%	2021	67.60%	2021	68.10%	2021	78.40%	2021	72.70%	2021	71%	2021	70.10%	2021	100%
Whether legal frameworks are in place to promote<br>equality and non-discrimination on the basis of sex	No Ish	2022	Yes	2022	Yes	2022	Yes	2022	Yes	2022	Yes	2022	Yes	2022	Yes	2022	Yes	2022	Yes	2022	100%
Whether legal frameworks are in place to promote<br>equality and non-discrimination on the basis of disability status	No	2022	In the works	2022	Yes	2022	Yes	2022	Yes	2022	Yes	2022	Yes	2022	Yes	2022	In the works	2022	Yes	2022	100%
Whether legal frameworks are in place to promote<br>equality and non-discrimination on the basis of sexual orientation	No	2022	No	2022	No	2022	No	2022	No	2022	No	2022	In the works	2022	No	2022	Yes	2022	In the works	2022	100%
Whether legal frameworks are in place to promote<br>equality and non-discrimination on the basis of religion	No	2022	Yes	2022	No	2022	Yes Ish	2022	Yes	2022	Yes	2022	Yes	2022	Yes	2022	Yes	2022	Yes	2022	100%
Human Freedom Index	5.62	2022	6.24	2022	6.74	2022	5.44	2022	6.78	2022	5.56	2022	6.52	2022	7.7	2022	6.49	2022	5.64	2022	100%
Level of national investment in medical research and basic health sectors	ND	ND	ND	ND	ND	ND	ND	ND	ND	ND	ND	ND	ND	ND	ND	ND	ND	ND	ND	ND	0%
																					**87.50%**

Contextual Factors: Economic Determinants

Country	Brunei Darussalam		Cambodia		Indonesia		Lao PDR		Malaysia		Myanmar		Philippines		Singapore		Thailand		Vietnam		
**Indicator**	**Value**	**Year**	**Value**	**Year**	**Value**	**Year**	**Value**	**Year**	**Value**	**Year**	**Value**	**Year**	**Value**	**Year**	**Value**	**Year**	**Value**	**Year**	**Value**	**Year**	**% Availability**
Gini coefficient	ND	ND	30.8	2012	37.9	2021	38.8	2018	41.1	2015	30.7	2017	42.3	2018	65.5	2019	35	2020	35.7	2018	90%
Multidimensional Poverty Index	ND	ND	0.17	2014	0.014	2017	0.108	2017	ND	ND	0.176	2015/2016	0.024	2017	ND	ND	0.002	2019	0	2020/2021	70%
State assistance for housing	Yes	2022	Yes	2022	Yes	2022	Yes	2022	Yes	2022	Yes	2022	Yes	2022	Yes	2022	Yes	2022	Yes	2022	100%
Presence of national rent control programmes (Y/N)	ND	ND	Yes	2022	No	2022	No	2022	No	2022	Yes	2022	Yes	2022	Yes	2022	Yes?	2022	No	2022	90%
Economic Policy Uncertainty Index score	ND	ND	ND	ND	ND	ND	ND	ND	ND	ND	ND	ND	ND	ND	271.51	2022	ND	ND	ND	ND	10%
Presence of national minimum wage	No	2022	Yes	2022	Yes	2022	Yes	2022	Yes	2022	Yes	2022	Yes	2022	No	2022	Yes	2022	Yes	2022	100%
Average working hours	44/week	2022	48/week	2022	40/week	2022	48/week	2022	45/week	2022	48/week	2022	43/week	2022	44.4/week	2021	48/week	2022	39/week	2021	100%
National minimum of paid leave days	12	2022	18	2022	12	2022	15	2022	16	2022	10	2022	12	2022	12	2022	6	2022	12	2022	100%
Percentage of informal workers nationally	8.10%	2019	93%	2022	59%	2021	93.60%	2018	10%	2021	85%	2022	56%	2022	10.40%	2022	55%	2022	56.20%	2021	100%
Poverty headcount ratio at national poverty lines (% of population)	ND	ND	17.7	2012	9.8	2020	18.3	2018	8.4	2019	24.8	2017	16.7	2018	ND	ND	6.8	2020	6.7	2018	80%
																					**84.00%**

Contextual Factors: Environmental Determinants

Country	Brunei Darussalam		Cambodia		Indonesia		Lao PDR		Malaysia		Myanmar		Philippines		Singapore		Thailand		Vietnam		
**Indicator**	**Value**	**Year**	**Value**	**Year**	**Value**	**Year**	**Value**	**Year**	**Value**	**Year**	**Value**	**Year**	**Value**	**Year**	**Value**	**Year**	**Value**	**Year**	**Value**	**Year**	**% Availability**
Crime																					
Global Organized Crime Index: Criminality Score	2.77	2022	5.83	2022	6.38	2022	5.52	2022	5.94	2022	7.59	2022	6.84	2022	3.13	2022	5.77	2022	6	2022	100%
Housing & Neighbourhood																					**17%**
Household overcrowding (%)	ND	ND	ND	ND	ND	ND	ND	ND	ND	ND	ND	ND	ND	ND	ND	ND	ND	ND	ND	ND	0%
Housing in poor condition (%)	ND	ND	ND	ND	ND	ND	ND	ND	ND	ND	ND	ND	ND	ND	ND	ND	ND	ND	ND	ND	0%
Exposure to asthma triggers	ND	ND	ND	ND	ND	ND	ND	ND	ND	ND	ND	ND	ND	ND	ND	ND	ND	ND	ND	ND	0%
Average distance to general store or supermarket	ND	ND	ND	ND	ND	ND	ND	ND	ND	ND	ND	ND	ND	ND	ND	ND	ND	ND	ND	ND	0%
Public transportation coverage	ND	ND	ND	ND	ND	ND	ND	ND	ND	ND	ND	ND	ND	ND	ND	ND	ND	ND	ND	ND	0%
Presence of parks or green areas	Yes	2022	Yes	2022	Yes	2022	Yes	2022	Yes	2022	Yes	2022	Yes	2022	Yes	2022	Yes	2022	Yes	2022	100%
Environmental Quality																					**53%**
Air Quality Index	14	2021	19.8	2021	34.3	2021	21.5	2021	19.4	2021	25.9	2021	15.6	2021	13.8	2021	20.2	2013	24.7	2021	100%
Household air quality (pm2.5 (µg/m3)) by capital city	ND	ND	ND	ND	46.35	2019	25.77	2019	28.27	2019	26.66	2017	29	2017	11	2020	23.14	2019	ND	ND	70%
No. of road traffic accidents involving injuries	1.69	2020	3.60%	2020	1.81%	2020	3.18%	2020	4.27%	2020	3.05%	2020	1.93	2020	0.51%	2020	5.04%	2020	4.30%	2020	100%
No. population living in proximity to major road/highway	ND	ND	ND	ND	ND	ND	ND	ND	ND	ND	ND	ND	ND	ND	ND	ND	ND	ND	ND	ND	0%
No. of population living in proximity to landfill	ND	ND	ND	ND	ND	ND	ND	ND	ND	ND	ND	ND	ND	ND	ND	ND	ND	ND	ND	ND	0%
No. of population living in proximity to factories	ND	ND	ND	ND	ND	ND	ND	ND	ND	ND	ND	ND	ND	ND	ND	ND	ND	ND	ND	ND	0%
Net change of tree cover from year 2000	-0.59%	2020	-26%	2020	-2.60%	2020	-7.20%	2020	-3.80%	2020	-3.50%	2020	-1.40%	2020	1.90%	2020	0.38%	2020	-3.10%	2020	100%
Water & Sanitation																					**84%**
People using safely managed drinking water services (% of population)	ND	ND	27.76	2020	ND	ND	17.68	2020	93.82	2020	58.83	2020	47.46	2020	100	2020	ND	ND	ND	ND	60%
People using at least basic drinking water services (% of population)	99.9	2020	71.22	2020	92.42	2020	85.22	2020	97.1	2020	83.72	2020	94.11	2020	100	2020	100	2020	96.88	2020	100%
People using safely managed sanitation services (% of population)	ND	ND	ND	ND	ND	ND	61.38	2020	77.45	2018	60.71	2020	60.64	2020	100	2020	25.93	2020	ND	ND	60%
People using at least basic sanitation services (% of population)	93.6	2015	68.77	2020	86.46	2020	79.48	2020	99.58	2018	73.64	2020	82.26	2020	100	2020	98.71	2020	89.25	2020	100%
Anthropogenic wastewater that receives treatment (%)	6.1	2018	0	2018	0	2018	0	2018	12.4	2018	0	2018	0.7	2018	100	2018	2	2018	0.3	2018	100%
Climate Change																					**100%**
Whether the country has communicated the establishment or operationalization of an integrated policy, strategy, or plan that increases its ability to adapt to the adverse impacts of climate change, and foster climate resilience and low greenhouse gas emissions development in a manner that does not threaten food production (including a national adaptation plan, nationally determined contribution, national communication, biennial update report or other)	Yes	2022	Yes	2022	Yes	2022	Yes	2022	Yes	2022	Yes	2022	Yes	2022	Yes	2022	Yes	2022	Yes	2022	100%

																					**54.50%**
**% Availability (53 indicators)**	**62.26%**		**71.70%**		**69.81%**		**75.47%**		**75.47%**		**75.47%**		**77.36%**		**71.70%**		**75.47%**		**69.81%**		**72.45%**
	33	53	38	53	37	53	40	53	40	53	40	53	41	53	38	53	40	53	37	53	
