# Supplementary material for: Measuring health equity in the ASEAN region: conceptual framework and assessment of data availability
Source: Int J Equity Health. 2023 Dec 5;22:251. doi: 10.1186/s12939-023-02059-2 (PMC10696689; doi:10.1186/s12939-023-02059-2)
Supplement: Supplementary file 4 — Supplementary Material 4 [file 12939_2023_2059_MOESM4_ESM.pdf]

Health System Input 1: Leadership and Governance

Country	Brunei Darussalam		Cambodia		Indonesia		Lao PDR		Malaysia		Myanmar		Philippines		Singapore		Thailand		Vietnam		
**Indicator**	**Value**	**Year**	**Value**	**Year**	**Value**	**Year**	**Value**	**Year**	**Value**	**Year**	**Value**	**Year**	**Value**	**Year**	**Value**	**Year**	**Value**	**Year**	**Value**	**Year**	**% Availability**
Average of 13 IHR Core Capacity Scores, SPAR Version	ND	ND	48	2020	69	2020	43	2020	86	2020	63	2020	69	2020	92	2020	85	2020	72	2020	90%
																					**90%**

Health System Input 2: Healthcare Information Systems

Country	Brunei Darussalam		Cambodia		Indonesia		Lao PDR		Malaysia		Myanmar		Philippines		Singapore		Thailand		Vietnam		
**Indicator**	**Value**	**Year**	**Value**	**Year**	**Value**	**Year**	**Value**	**Year**	**Value**	**Year**	**Value**	**Year**	**Value**	**Year**	**Value**	**Year**	**Value**	**Year**	**Value**	**Year**	**% Availability**
No. of MOH/public hospitals that have implemented HIS or EMR	100%	2022	20	2022	mandated, but ~80% have not	2022	at least 4	2022	37	2020	ND	ND	at least 2000	2016	1908	2022	116	2019	100%	2019	90%
No. of MOH/public primary health clinics that have implemented EMR	100%	2022		2022		2022	ND	ND	97	2020	at least 25	2020	at least 3000	2016		2022	ND	ND	ND	ND	70%
National system of data collection based on health service delivery	Yes	Bru-HIMS	No	2016	No	2016	No	2016	Yes	2016	Yes	2016	No	2016	Yes	2016	Yes	2016	No	2016	100%
Presence of a centralized data warehouse to provide linkages across various health and non-health datasets	Yes	2022	ND	ND	No	2022	Yes	2017	Yes	2022	ND	ND	Yes	2022	Yes	2022	ND	ND	ND	ND	60%
Completeness of cause-of-death data	99.67	2016	ND	ND	ND	ND	ND	ND	51.81	2014	ND	ND	88.95	2011	66.12	2016	87.23	2016	ND	ND	50%
																					**74%**

Health System Input 3: Health Financing

Country	Brunei Darussalam		Cambodia		Indonesia		Lao PDR		Malaysia		Myanmar		Philippines		Singapore		Thailand		Vietnam		
**Indicator**	**Value**	**Year**	**Value**	**Year**	**Value**	**Year**	**Value**	**Year**	**Value**	**Year**	**Value**	**Year**	**Value**	**Year**	**Value**	**Year**	**Value**	**Year**	**Value**	**Year**	**% Availability**
Current Health Expenditure																					**100%**
Current health expenditure (CHE) as percentage of gross domestic product (GDP) (%)	2.16	2019	6.99	2019	2.9	2019	2.6	2019	3.83	2019	4.68	2019	4.08	2019	4.08	2019	3.79	2019	5.25	2019	100%
Current health expenditure (CHE) per capita in PPP int$	1401	2019	316.1	2019	358.3	2019	212.4	2019	1133	2019	226.8	2019	379.2	2019	4102	2019	731.3	2019	558.9	2019	100%
Current health expenditure (CHE) per capita in US$	671.6	2019	113.3	2019	120.1	2019	68.22	2019	436.6	2019	60.02	2019	142.1	2019	2633	2019	296.2	2019	180.7	2019	100%
Out-of-Pocket Expenditure																					**100%**
Out-of-pocket expenditure as percentage of current health expenditure (CHE) (%)	5.68	2019	64.39	2019	34.76	2019	41.83	2019	34.57	2019	75.95	2019	48.56	2019	30.15	2019	8.67	2019	42.95	2019	100%
Out-of-pocket expenditure (OOP) per capita in PPP int$	79.57	2019	203.5	2019	124.6	2019	88.84	2019	391.7	2019	172.3	2019	184.2	2019	1237	2019	63.4	2019	240.1	2019	100%
Out-of-pocket expenditure (OOP) per capita in US$	38.15	2019	72.96	2019	41.76	2019	28.54	2019	151	2019	45.58	2019	68.99	2019	793.8	2019	25.68	2019	77.62	2019	100%
Domestic General Government Health Expenditure																					**100%**
Domestic general health expenditure (GCHE-D) as % of general gov expenditure (GGE)	6.81	2019	7.04	2019	8.68	2019	4.71	2019	8.48	2019	3.64	2019	7.63	2019	14.54	2019	13.87	2019	10.07	2019	100%
Domestic general government health expenditure (GGHE-D) as percentage of gross domestic product (GDP) (%)	2.04	2019	1.7	2019	1.42	2019	0.96	2019	2	2019	0.74	2019	1.66	2019	2.05	2019	2.72	2019	2.3	2019	100%
Domestic general government health expenditure (GGHE-D) per capita in PPP int$	1321	2019	76.82	2019	175.3	2019	78.44	2019	591.4	2019	35.74	2019	154	2019	2059	2019	524.1	2019	244.8	2019	100%
Domestic general government health expenditure (GGHE-D) per capita in US$	633.4	2019	27.54	2019	58.79	2019	25.2	2019	227.9	2019	9.46	2019	57.68	2019	1322	2019	212.2	2019	79.15	2019	100%
Domestic Private Health Expenditure																					**100%**
Domestic private health expenditure (PVT-D) as percentage of current health expenditure (CHE) (%)	5.68	2019	69.19	2019	50.51	2019	41.86	2019	47.8	2019	75.96	2019	58.99	2019	49.8	2019	28.23	2019	55.23	2019	100%
Domestic private health expenditure (PVT-D) per capita in PPP int$	79.57	2019	218.7	2019	181	2019	88.89	2019	541.6	2019	172.3	2019	223.7	2019	2043	2019	206.4	2019	308.7	2019	100%
Domestic private health expenditure (PVT-D) per capita in US$	38.15	2019	78.4	2019	60.67	2019	28.56	2019	208.7	2019	45.59	2019	83.81	2019	1311	2019	83.6	2019	99.81	2019	100%
External Health Expenditure																					**100%**
External health expenditure (EXT) as percentage of current health expenditure (CHE) (%)	0	2019	6.5	2019	0.56	2019	21.21	2019	0	2019	8.29	2019	0.41	2019	0	2019	0.11	2019	0.97	2019	100%
External health expenditure (EXT) per capita in PPP int$	0	2019	20.56	2019	1.99	2019	45.04	2019	0	2019	18.79	2019	1.57	2019	0	2019	0.83	2019	5.42	2019	100%
External health expenditure (EXT) per capita in US$	0	2019	7.37	2019	0.67	2019	14.47	2019	0	2019	4.97	2019	0.59	2019	0	2019	0.34	2019	1.75	2019	100%
																					**100%**

Health System Input 4: Health Workforce

Country	Brunei Darussalam		Cambodia		Indonesia		Lao PDR		Malaysia		Myanmar		Philippines		Singapore		Thailand		Vietnam		
**Indicator**	**Value**	**Year**	**Value**	**Year**	**Value**	**Year**	**Value**	**Year**	**Value**	**Year**	**Value**	**Year**	**Value**	**Year**	**Value**	**Year**	**Value**	**Year**	**Value**	**Year**	**% Availability**
Medical Doctors																					**81.67%**
Medical doctors (number)	683	2017	2944	2014	170338	2020	2575	2020	73973	2020	39826	2019	84678	2020	14279	2019	66301	2020	77539	2016	100%
Doctors per 1,000 population (OECD/WHO)	1.6	latest available	0.2	latest available	0.4	latest available	0.4	latest available	1.5	latest available	0.7	latest available	0.6	latest available	2.3	latest available	0.8	latest available	0.8	latest available	100%
Medical doctors (per 10,000 population)	16.09	2017	1.93	2014	6.23	2020	3.54	2020	22.86	2020	7.37	2019	7.73	2020	24.6	2019	9.5	2020	8.28	2016	100%
Generalist medical practitioners (number)	262	2014	ND	ND	137920	2020	ND	ND	ND	ND	ND	ND	ND	ND	7920	2016	66301	2020	ND	ND	40%
Specialist medical practitioners (number)	357	2014	376	2014	32418	2020	ND	ND	ND	ND	ND	ND	ND	ND	5047	2016	13492	2016	ND	ND	50%
Medical numbers not further defined (number)	683	2017	2568	2014	0	2020	2575	2020	73973	2020	39826	2019	84678	2020	14279	2019	63974	2019	77539	2016	100%
Nursing and Midwifery Personnel																					**100%**
Nursing personnel (number)	2530	2018	9483	2019	615829	2020	6981	2020	111324	2019	40830	2019	512719	2019	35636	2017	219473	2019	106654	2016	100%
Nurses and midwives (per 1,000 people)	5.9	2018	1	2019	3.8	2019	0.7	2019	3.5	2019	1.1	2019	5.4	2019	6.2	2017	3.2	2019	1.4	2016	100%
Nursing and midwifery personnel (per 10 000 population)	58.97	2018	10.08	2019	39.54	2020	11.9	2020	34.84	2019	10.82	2019	54.44	2019	62.43	2017	31.52	2019	14.46	2016	100%
Dentistry Personnel																					**90%**
Dentists (number)	110	2019	1385	2018	32418	2020	559	2020	9717	2018	3800	2019	28154	2019	2363	2018	18560	2019	ND	ND	90%
Dentists (per 10 000 population)	2.54	2019	0.85	2018	1.19	2020	0.77	2020	3.08	2018	0.7	2019	2.6	2019	4.1	2018	2.67	2019	ND	ND	90%
Dental Assistants and Therapists (number)	101	2019	383	2018	22883	2020	91	2018	2928	2018	344	2012	246	2019	389	2018	17676	2018	ND	ND	90%
Pharmaceutical Personnel																					**93.33%**
Pharmacists (number)	71	2015	526	2014	25625	2020	1664	2020	10511	2015	4203	2019	34767	2017	2875	2016	43936	2019	31719	2016	100%
Pharmacists (per 10 000 population)	1.71	2015	0.34	2014	0.94	2020	2.29	2020	3.47	2015	0.78	2019	3.31	2017	5.09	2016	6.31	2019	3.39	2016	100%
Pharmaceutical Technicians and Assistants (number)	116	2012	89	2014	36026	2020	ND	ND	5308	2015	3762	2015	25000	2011	ND	ND	8004	2016	1278	2016	80%
Others																					**63.75%**
Environmental and Occupational Health and Hygiene Professionals (number)	91	2015	7123	2012	409	2020	985	2014	4952	2012	3748	2015	2737	2015	ND	ND	1117	2016	ND	ND	80%
Environmental and Occupational Health Inspectors and Associates (number)	350	2014	ND	ND	26508	2020	ND	ND	4517	2015	10716	2018	ND	ND	ND	ND	7265	2016	ND	ND	50%
Medical and Pathology Laboratory Scientists (number)	113	2015	554	2014	0	2019	636	2014	6161	2012	1596	2019	ND	ND	ND	ND	ND	ND	ND	ND	60%
Medical and Pathology Laboratory Technicians (number)	27	2015	531	2012	59818	2020	460	2012	6324	2015	4501	2019	ND	ND	ND	ND	2442	2016	ND	ND	70%
Physiotherapists (number)	21	2015	176	2014	15156	2020	252	2014	1361	2015	118	2004	ND	ND	1693	2016	4355	2018	ND	ND	80%
Physiotherapy Technicians and Assistants (number)	19	2012	ND	ND	0	2019	ND	ND	ND	ND	ND	ND	ND	ND	ND	ND	42282	2016	ND	ND	30%
Traditional and Complementary Medicine Professionals (number)	ND	ND	317	2013	595	2020	3128	2010	12226	2010	1255	2019	ND	ND	2868	2016	2113	2016	12948	2009	80%
Community Health Workers	ND	ND	1638	2004	45709	2020	75	2019	ND	ND	2326	2015	248584	2019	ND	ND	39928	2019	ND	ND	60%
																					**84.42%**

Health System Input 5: Access to Essential Medicine

Country	Brunei Darussalam		Cambodia		Indonesia		Lao PDR		Malaysia		Myanmar		Philippines		Singapore		Thailand		Vietnam		
**Indicator**	**Value**	**Year**	**Value**	**Year**	**Value**	**Year**	**Value**	**Year**	**Value**	**Year**	**Value**	**Year**	**Value**	**Year**	**Value**	**Year**	**Value**	**Year**	**Value**	**Year**	**% Availability**
Median availability of selected generic medicines (%) - Public	ND	ND	ND	ND	65.5	2007-2013	ND	ND	ND	ND	ND	ND	26.8	2007-2013	ND	ND	ND	ND	ND	ND	20%
Median availability of selected generic medicines (%) - Private	ND	ND	ND	ND	57.8	2007-2013	ND	ND	ND	ND	ND	ND	21.7	2007-2013	ND	ND	ND	ND	ND	ND	20%

Proportion of health facilities with a core set of relevant essential medicines available and affordable on a sustainable basis	ND	ND	ND	ND	14.65	2010	25.27	2013	ND	ND	ND	ND	ND	ND	ND	ND	ND	ND	ND	ND	20%
																					**20%**

Health System Input 5: Access to Essential Medicine

Country	Brunei Darussalam		Cambodia		Indonesia		Lao PDR		Malaysia		Myanmar		Philippines		Singapore		Thailand		Vietnam		
**Indicator**	**Value**	**Year**	**Value**	**Year**	**Value**	**Year**	**Value**	**Year**	**Value**	**Year**	**Value**	**Year**	**Value**	**Year**	**Value**	**Year**	**Value**	**Year**	**Value**	**Year**	**% Availability**
No. of primary health clinic	17	2021	244	2015	10100	2020	1050	2019	1,138	2020	5,131	2015	ND	ND	2,363	2020	25,000	2022	35,593	2015	90%
No. of clinics per 1,000 population	ND	ND	ND	ND	ND	ND	ND	ND	ND	ND	ND	ND	ND	ND	ND	ND	ND	ND	ND	ND	0%
No. of hospitals - Public	4	2021	126	2022	1,026	2021	195	2017	156	2020	1120	2016+	721	2019	16	2022	1,047	2017	1150	2022	100%
No. of hospitals - Private	3	2021	54	2016	1,787	2018	17	2019	219	2020	249	2016+	1071	2019	7	2022	370	2020	306	2021	100%
Total density per 100 000 population: Hospitals	1.44	2013	0.57	2013	0.43	2013	2.25	2013	0.47	2013	0.63	2013	1.81	2013	0.5	2013	1.84	2013	ND	ND	90%
Hospital beds - per 1,000 population	2.9	2017	0.9	2016	1	2017	1.5	2012	1.9	2017	1	2017	1	2014	2.5	2017	2.1	2010	2.6	2014	100%
Hospital beds - per 10,000 population	28.5	2017	9	2016	10.4	2017	15	2012	18.77	2017	10.44	2017	9.9	2014	24.86	2017	21	2005	31.8	2013	100%
Beds in community residential facilities (per 100,000 population)	2.874	2016	ND	ND	ND	ND	ND	ND	ND	ND	ND	ND	2.045	2016	6.431	2016	ND	ND	ND	ND	30%
Beds in mental hospitals (per 100,000 population)	0	2016	ND	ND	ND	ND	ND	ND	11.054	2016	2.672	2016	4.129	2016	34.867	2016	6.464	2016	8.103	2016	70%
% of acute beds in public secondary and tertiary healthcare (STHC)	ND	ND	ND	ND	ND	ND	ND	ND	ND	ND	ND	ND	ND	ND	ND	ND	ND	ND	ND	ND	0%
% of acute beds in pte STHC	ND	ND	ND	ND	ND	ND	ND	ND	ND	ND	ND	ND	ND	ND	ND	ND	ND	ND	ND	ND	0%
Medical devices (density per million population of selected medical devices in public & pte facilities) - Computed Tomography	7.18	2013	1.19	2013	ND	ND	0.74	2013	6.43	2013	0.075	2013	1.09	2013	8.87	2013	5.95	2013	ND	ND	80%
Medical devices (density per million population of selected medical devices in public & pte facilities) - Radiotherapy	ND	ND	0.066	2013	0.15	2013	0	2013	1.41	2013	0.056	2013	0.18	2013	3.51	2013	0.97	2013	0.4	2013	90%
Medical devices (density per million population of selected medical devices in public & pte facilities) - MRI	2.39	2013	0.066	2013	ND	ND	0	2013	2.89	2013	0.075	2013	0.3	2013	7.76	2013	ND	ND	ND	ND	70%
Medical devices (density per million population of selected medical devices in public & pte facilities) - Ventilators	ND	ND	ND	ND	ND	ND	ND	ND	ND	ND	ND	ND	ND	ND	ND	ND	ND	ND	ND	ND	0%
Medical devices (density per million population of selected medical devices in public & pte facilities) - Mammographs	91.93	2014	ND	ND	ND	ND	0	2014	86.7	2014	0.71	2014	13.12	2014	127.6	2014	27.87	2014	ND	ND	70%
																					**61.88%**

**% Availability (64 indicators)**	**82.81%**		**76.56%**		**84.38%**		**76.56%**		**81.25%**		**78.13%**		**78.13%**		**78.13%**		**81.25%**		**57.81%**		**77.50%**
	53	64	49	64	54	64	49	64	52	64	50	64	50	64	50	64	52	64	37	64	
